# Systems biology modeling of omics data: effect of cyclosporine a on the Nrf2 pathway in human renal cells

**DOI:** 10.1186/1752-0509-8-76

**Published:** 2014-06-25

**Authors:** Jérémy Hamon, Paul Jennings, Frederic Y Bois

**Affiliations:** 1 Mathematical Modeling for Systems Toxicology, Université de Technologie de Compiègne, BP 20529, 60205 Compiègne Cedex, France; 2 Division of Physiology, Department of Physiology and Medical Physics, Innsbruck Medical University, Innsbruck 6020, Austria; 3 INERIS, DRC/VIVA/METO, Parc ALATA, BP 2, 60550 Verneuil en Halatte, France

**Keywords:** Bayesian data analysis, Cyclosporine A, Glutathione pathway, Integrated omics, Nrf2 pathway, Oxidative stress, Renal proximal tubule epithelial cells, Systems biology

## Abstract

**Background:**

Incorporation of omic data streams for building improved systems biology models has great potential for improving their predictions of biological outcomes. We have recently shown that cyclosporine A (CsA) strongly activates the nuclear factor (erythroid-derived 2)-like 2 pathway (Nrf2) in renal proximal tubular epithelial cells (RPTECs) exposed *in vitro*. We present here a quantitative calibration of a differential equation model of the Nrf2 pathway with a subset of the omics data we collected.

**Results:**

*In vitro* pharmacokinetic data on CsA exchange between cells, culture medium and vial walls, and data on the time course of omics markers in response to CsA exposure were reasonably well fitted with a coupled PK-systems biology model. Posterior statistical distributions of the model parameter values were obtained by Markov chain Monte Carlo sampling in a Bayesian framework. A complex cyclic pattern of ROS production and control emerged at 5 μM CsA repeated exposure. Plateau responses were found at 15 μM exposures. Shortly above those exposure levels, the model predicts a disproportionate increase in cellular ROS quantity which is consistent with an *in vitro* EC_50_ of about 40 μM for CsA in RPTECs.

**Conclusions:**

The model proposed can be used to analyze and predict cellular response to oxidative stress, provided sufficient data to set its parameters to cell-specific values. Omics data can be used to that effect in a Bayesian statistical framework which retains prior information about the likely parameter values.

## Background

The quantitative modeling of toxicity pathways is a topic of current interest in predictive pharmacology and toxicology [1-3]. One of its challenges is to integrate omics data with systems biology models for parametric inference and model checking [4]. In a recent paper, Wilmes *et al.*[5] demonstrated a qualitative integration of transcriptomic (TCX), proteomic (PTX) and metabolomic (MTX) data streams to gain a mechanistic understanding of cyclosporine A (CsA) toxicity. CsA is an important molecule for immunosuppressive treatment and is used in many post-graft medical protocols [6]. It is mainly metabolized by CYP3A4 and CYP3A5 and is a substrate of the P-glycoprotein (ABCB1) efflux transporter [6,7]. However, at high dose it is nephrotoxic and causes damage to the kidney vasculature, glomerulus and proximal tubule [8-10]. CsA is thought to induce oxidative stress at the mitochondrial level, and co-administration of antioxidants with CsA appears to mitigate its nephrotoxic effects [11], yet, the precise mechanisms of its toxicity are still unclear.

The Nrf2 oxidative response pathway is triggered when oxidative stress is sensed by Keap-1, resulting in stabilization and nuclear translocation of Nrf2 [12]. Nrf2 binds to the antioxidant response element (ARE) inducing the transcription of several genes involved in glutathione synthesis and recycling, antioxidant activity, phase II metabolism and transport [12]. The Nrf2 response has been shown to be induced in several tissues in response to chemical or physiological stress. The kidney and particularly the proximal tubule is especially sensitive to oxidative stress. Many nephrotoxins induce Nrf2 nuclear translocation and Nrf2-dependent gene induction in renal epithelial cells, including potassium bromate, cadmium chloride, diquat dibromide and cyclosporine A [5,13,14]. Moreover, we have recently shown that physiological stress such as glucose depletion and subsequent re-introduction results in Nrf2 activation in renal cells [15]. Here, we use a subset of the Wilmes’ *et al.*[5] omics data to calibrate the parameters of a systems biology model describing the Nrf2 pathway. The model predictive ability is then assessed by comparison to CsA toxicity data on RPTEC cells.

## Methods

### Data

RPTECs culture conditions, CsA concentrations measurements, and TCX, PTX and MTX data collection and analysis were described in detail in Wilmes *et al.*[5]. Briefly, RPTECs cells were cultured in 3 ml of serum-free medium and matured for two weeks on microporous supports. They were then treated for fourteen days with daily doses of CsA. The assay medium was renewed prior to each dosing. Three groups of assays were performed in triplicate: control, low CsA concentration (5 μM) and high CsA concentration (15 μM).

CsA concentration was measured in the medium on the first day at 0.5 h, 1 h, 3 h, 6 h and 24 h (just before changing the medium), on the third, fifth, seventh, and tenth day at 24 h (before changing the medium), and on day fourteenth at the same times than on the day one. Intracellular (cell lysate) CsA concentration and quantity bound to plastic were measured on the first and last days at the same times. Samples for TCX (on Illumina® HT 12 v3 BeadChip arrays), PTX (obtained by HPLC-MS) and MTX (by direct infusion MS) measurements were collected at the end of day 1, day 3 and day 14. All fold-changes were calculated using the absolute value measured at the first time of the control experiment as a reference, and for all doses. Typical RPTEC cell volume was determined by electron microscopy and stereology to be 2000 ± 140 μm^3^. On average, 2.1 millions cells were present in each assay well. All those data are given as additional material (Additional file 1: Tables S2 to S9).

### Mathematical models

**Figure 1:**
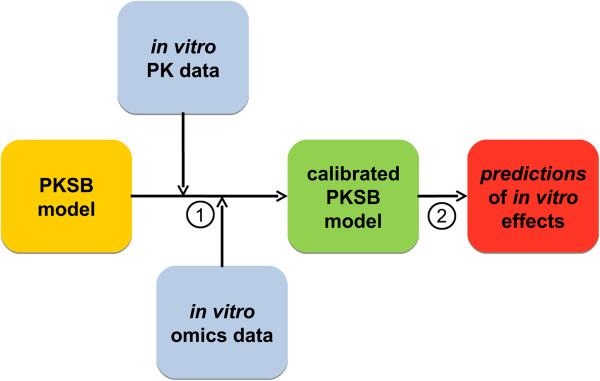
**Schematic representation of the calibration (1) and prediction (2) processes used in this article.** The coupled pharmacokinetic-systems biology model (PKSB) of the Nrf2 pathway was calibrated by MCMC sampling in a Bayesian framework with PK and omics data obtained during repeated treatment of RPTECs by CsA. After calibration, the model was used to make predictions enabling model checking.

Modeling was done into two steps: (i) Modeling the *in vitro* pharmacokinetics (PK) of CsA (exchange between cells, medium and vial walls) with a minimal distribution model. (ii) Modeling the effects of CsA on omics markers at the cellular level with a coupled PK-systems biology model. That model was calibrated by Markov chain Monte Carlo (MCMC) sampling [4] with the above *in vitro* PK and omics data used together. Calibration summarizes and integrates the information brought by the various types of omics data into the coherent scheme of a unified model. The calibrated model was then run to predict various quantities of interest at a higher level in the hierarchy of biological effects. Such predictions can be compared to further observations, for example on toxicity, to help check the model. That process is shown schematically on Figure 1.

*In vitro pharmacokinetic model.* A 3-compartment model was developed to describe CsA exchange between cell medium, cells and vial walls [5]. In that model, CsA can enter and exit the cells, bind to and unbind from the plastic walls and can be metabolized within cells. Several mathematical forms for exchange rates were tested. The best fit was obtained using a first order entry into cells with Michaelis-Menten (saturable) exit rate, a first order attachment to vial wall with non-integer (fractal) order detachment, and Michaelis-Menten metabolism. The following differential equations were used to describe the time course of CsA quantities in the cytosol, medium, and on vial walls:

(1) $$\begin{array}{ll} \frac{\partial \mathrm{Cs}A_{\mathrm{cytosol}}}{\partial t}= & CL_{in_{1}}\frac{\mathrm{Cs}A_{\mathrm{extracellular}}}{V_{\mathrm{extracellular}}}\\ & -\frac{CL_{\mathrm{ou}t_{1}}\cdot \mathrm{Cs}A_{\mathrm{cytosol}}}{V_{\mathrm{cytosol}}\cdot Km_{\mathrm{ou}t_{1}}+\mathrm{Cs}A_{\mathrm{cytosol}}}\\ & -\frac{v_{\max}\cdot \mathrm{Cs}A_{\mathrm{cytosol}}}{Km_{2}+\mathrm{Cs}A_{\mathrm{cytosol}}} \end{array}$$

(2) $$\begin{array}{ll} \frac{\partial \mathrm{Cs}A_{\mathrm{extracellular}}}{\partial t}= & -CL_{in_{1}}\frac{\mathrm{Cs}A_{\mathrm{extracellular}}}{V_{\mathrm{extracellular}}}\\ & +\frac{CL_{\mathrm{ou}t_{1}}\cdot \mathrm{Cs}A_{\mathrm{cytosol}}}{V_{\mathrm{cytosol}}\cdot Km_{\mathrm{ou}t_{1}}+\mathrm{Cs}A_{\mathrm{cytosol}}}\\ & -k_{1}\cdot \mathrm{Cs}A_{\mathrm{extracellular}}+k_{2}{\left(\mathrm{Cs}A_{\mathrm{wall}}\right)}^{k_{3}} \end{array}$$

(3) $$\frac{\partial \mathrm{Cs}A_{\mathrm{wall}}}{\partial t}=k_{1}\cdot \mathrm{Cs}A_{\mathrm{extracellular}}-k_{2}{\left(\mathrm{Cs}A_{\mathrm{wall}}\right)}^{k_{3}}$$

The model parameters are described in Table 1.

**Table 1:** ***In vitro *** **CsA kinetic parameters description and their statistical distributions**

**Parameter**	**Description**	**Units**	**Prior**	**Posterior mode, mean ± SD**
$CL_{in_{1}}$	Diffusion rate constant for cellular uptake	μm^3^.sec^−1^	LU*(10^−1^, 104)	99.6,
				99.8 ± 21
$Km_{\mathrm{ou}t_{1}}$	Michaelis constant for diffusion for cellular efflux	μmol.L^−1^	LU (100, 50000)	2965,
				3160 ± 620
$\frac{CL_{\mathrm{ou}t_{1}}}{Km_{\mathrm{ou}t_{1}}}$	Diffusion rate constant over Michaelis constant for cellular efflux	μm^3^.sec^−1^	LU (10^−2^, 20)	0.581,
				0.568 ± 0.16
*k*_1_	Plastic binding rate constant	sec^−1^	LU (10^−6^, 5 × 10^−4^)	3.55 × 10^−5^,
				3.54 × 10^−5^ ± 1.0 × 10^−5^
*k*_3_	Power law coefficient for unbinding	dimensionless	Uniform (0, 0.95)	0.921,
				0.802 ± 0.074
*k*_2_	Plastic unbinding rate constant	zmol^(1-k3)^**.sec^−1^	LU (10^−4^, 0.5)	6.01 × 10^−4^,
				6.09 × 10^−3^ ± 8.7 × 10^−3^
*v*_*max*_	Maximum rate of metabolism	zmol.sec^−1^	LU (0.1, 5000)	40.0,
				47.2 ± 14
$K_{m_{2}}$	Michaelis constant for intra-cellular metabolism	zmol	LU (5 × 10^5^, 5 × 10^7^)	2.18 × 10^6^,
				3.43 × 10^6^ ± 2.2 × 10^6^

^*^: *LU*: Log-uniform distribution (lower bound, upper bound).

^**^: 1 zmol = 1 zeptomole = 10^−21^ mol.

**Figure 2:**
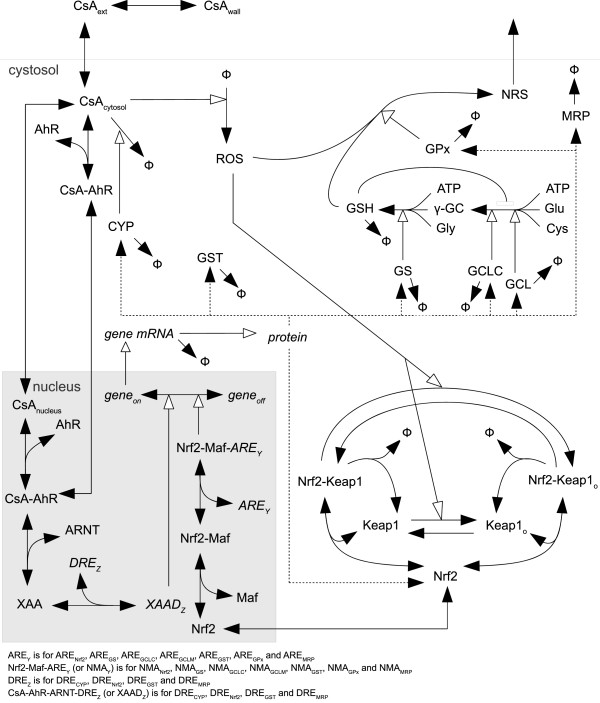
Schematic representation of the coupled pharmacokinetic-systems biology model of the Nrf2 pathway.

*Coupled PK-systems biology model of the Nrf2 pathways* (Figure 2)*.* The model used was adapted from Zhang *et al.*[16]. The model full set of equations is provided in the Additional file 1: Section 1. In brief, CsA induces oxidative stress by increasing reactive oxygen species (ROS) production. ROS, owing to their electrophilicity, can be detected by the molecular sensor Kelch-like ECH-associated protein 1 (Keap1), which promotes the ubiquitination and eventual degradation of Nrf2 [17,18]. When Keap1 is oxidized, Nrf2 ubiquitination is lowered [18], making Nrf2 available to enter the nucleus. Once in the nucleus, Nrf2 binds to small Maf proteins to form Nrf2-Maf heterodimers [19]. Those can bind to antioxidant responses elements (ARE) in the promoter region of glutamate cysteine ligase catalytic subunit (GCLC), glutamate cysteine ligase modifier subunit (GCLM), glutathione synthetase (GS), glutathione peroxidase (GPx), and ABCC2 genes [17,19], Maher, 2007 #27. GCLC, GCLM, and GS are involved in GSH synthesis. GPx detoxifies ROS, using GSH as a co-substrate. Zhang’s model was developed for a generic xenobiotic, so the following structural changes were made to consistently describe the cell kinetics and mode of action of CsA:

– CsA can enter or exit the cell, and attach to or detach from the vial walls as in the *in vitro* PK model (eqs. A10, A11, A13). Inside the cell, CsA distribution to the nucleus is also modeled (eq. A12);

– In the cell, CsA is metabolized by cytochrome P450 3A5 (CYP3A5) into a metabolite X’ (not followed because without influence on the model components). CsA is mainly metabolized by CYP3A isoforms [6], and in kidney cells *in vivo* only CYP3A5 is significantly expressed [20];

– Oxidative stress, characterized by the total quantity of oxidative compounds in the cell (ROS), was explicitly introduced in the model as a state variable (eq. A75);

– The production of ROS depends on CsA concentration in the cell, and ROS are eliminated by GPx (eq. A75) in a non-reactive species pool (NRS) (not followed and non-influent on the system);

– Keap1 and the Nrf2-Keap1 complex are oxidized by ROS (eqs. A52, A53, A72, A73).

All the other equations are the same as in Zhang *et al.*[16]. In addition, some parameters had to be set to particular values for CsA: In the model, xenobiotics can bind to the AhR nuclear receptor. However, CsA is not a known AhR ligand, so its binding parameters (*k*_*b*2_ and *k*_*b*5_) were set to zero. Additional file 1: Table S1 gives the model parameters’ values and the state variables’ initial values.

### Calibration of the models

Bayesian inference [4,21] was used to calibrate all the *in vitro* PK model parameters with data on the CsA quantities measured in the medium, cells, and on vial walls (Additional file 1: Table S2 to S7). Non-informative (vague uniform) prior parameter distributions were used (see Table 1). Their bounds were set to span an arbitrarily large range of values without constraining estimation. The data likelihoods were assumed to follow a lognormal distribution around the model predictions, a standard assumption with such measurements. Measurement errors’ geometric standard deviations were assumed to be specific of each of the three measurement types (different procedure were used for their obtention). They were assigned vague log-uniform prior distributions (spanning a range corresponding to approximate coefficients of variation from 1% to a factor 2) and were calibrated together with the other model parameters. Therefore a total of 11 parameters (8 kinetic parameters and 3 statistical ones) were calibrated. The posterior statistical distributions of those parameters were obtained by MCMC sampling [4].

For the coupled PK-Nrf2 pathway model, the parameters directly controlling CsA kinetics were set to the joint posterior distribution mode found by the above calibration (see Table 1). Another 27 structural parameters (Table 2) were selected for calibration on the basis of our understanding of the model structure with the help of a preliminary Monte Carlo sensitivity analysis [22] (see Additional file 1: Section 2). They were calibrated using fold-change omics data (as a function of time and CsA dose) on Nrf2 mRNA, CYP3A5 mRNA, GS mRNA, GCLC mRNA, GCLM mRNA, GST mRNA, GPx mRNA, ABCC2 mRNA, GCLM protein, GS protein, MRP2 protein, γ-glutamylcysteine (γ-GC), and GSH. Four of the parameters calibrated have a direct influence on the rate of ROS synthesis, metabolism and interaction with Keap1. Another 15 parameters control the activation and induction of Nrf2, GCLC, GCLM, GST, GPx, CYP3A5, GS and ABCC2 genes transcription. Another six parameters control synthesis and degradation of γ-GC and GSH, and the two last parameters control CsA metabolism and Nrf2 and Maf binding. Model predicted fold-changes were computed the same way as the experimental ones, using the quantity predicted at the first time of the control experiment as a reference. The prior parameter distributions chosen were either vague uniform distributions (spanning 6 orders of magnitude) or lognormal distributions centered around the values used by Zhang [16] with a geometric SD corresponding to a factor 3 (Table 2). The data likelihoods were assumed to be lognormal distributions around the model predictions. The same measurement error geometric standard deviation was assumed for all omics measurements. It was calibrated together with the other model parameters, using a vague log-uniform prior. Here also, posterior distributions of the parameter values were obtained by MCMC sampling. For each model parameter sampled, convergence was evaluated by computing the potential scale reduction criterion of Gelman and Rubin [23].

**Table 2:** Systems biology model parameters description and their statistical distributions

**Parameter**	**Description**	**Units**	**Prior distribution**	**Posterior mode, mean ± SD**
$v_{\mathrm{ma}x_{7}}$	Maximum rate of CsA metabolism	sec^−1^	LN (0.2, 3)	0.187
				0.274 ± 0.233
$k_{f_{75}}$	Basal rate of ROS formation	zmol.sec^−1^	LN (12, 3)	79.1
				135 ± 80.8
$v_{\mathrm{ma}x_{8b}}$	Maximum rate of ROS metabolism	sec^−1^	LN (8, 3)	2.67
				3.88 ± 2.54
$k_{ox_{10}}$	Keap1 oxidation rate constant	zmol^−1^.sec^−1^	Uniform (10^−8^, 10^−2^)	3.02 × 10^−6^
				3.86 × 10^−6^ ± 2.72 × 10^−6^
*k*_*ROS*_	ROS formation rate constant	sec^−1^	Uniform (10^−8^, 10^−2^)	6.55 × 10^−5^
				8.86 × 10^−6^ ± 3.86 × 10^−5^
$k_{b_{18}}$	Nrf2 and Maf binding rate constant	sec^−1^	LN (0.003, 3)	0.0124
				0.0193 ± 0.0167
$k_{\mathrm{TS}P_{21}}$	mRNA_CYP_ transcription rate constant	sec^−1^	LN (1.07, 3)	1.29
				1.65 ± 1.85
$k_{\mathrm{TS}P_{28}}$	mRNA_Nrf2_ transcription rate constant	sec^−1^	LN (0.00611, 3)	0.087
				0.062 ± 0.0603
$k_{\mathrm{TS}P_{34}}$	mRNA_GS_ transcription rate constant	sec^−1^	LN (1.15, 3)	1.07
				1.34 ± 0.53
$k_{\mathrm{TS}P_{42}}$	mRNA_GCLC_ transcription rate constant	sec^−1^	LN (1.98, 3)	1.28
				2.27 ± 1.91
$k_{\mathrm{TS}P_{48}}$	mRNA_GCLM_ transcription rateconstant	sec^−1^	LN (3.22, 3)	3.95
				4.84 ± 3.79
$k_{\mathrm{TS}P_{57}}$	mRNA_GST_ transcription rate constant	sec^−1^	LN (0.242, 3)	0.021
				0.553 ± 0.949
$k_{\mathrm{TS}P_{57b}}$	mRNA_GPx_ transcription rate constant	sec^−1^	LN (0.242, 3)	0.098
				0.123 ± 0.0779
$k_{\mathrm{TS}P_{66}}$	mRNA_MRP_ transcription rate constant	sec^−1^	LN (0.9, 3)	1.22
				2.23 ± 3.55
$k_{b_{52}}$	GCLC and GCLM binding rate constant	sec^−1^	LN (2 × 10^−5^, 3)	4.33 × 10^−6^
				1.09 × 10^−5^ ± 9.19 × 10^−6^
$k_{\mathrm{ind}{\left(\mathrm{NMA}\right)}_{27}}$	Induction coefficient for Nrf2 gene	zmol^−1^.sec^−1^	LN (100, 3)	150
				236 ± 433
$k_{\mathrm{ind}{\left(\mathrm{NMA}\right)}_{33}}$	Induction coefficient for GS gene	zmol^−1^.sec^−1^	LN (5.95, 3)	2.17
				3.85 ± 2.33
$k_{\mathrm{ind}{\left(\mathrm{NMA}\right)}_{41}}$	Induction coefficient for GCLC gene	zmol^−1^.sec^−1^	LN (8.7, 3)	22.1
				43.2 ± 25
$k_{\mathrm{ind}{\left(\mathrm{NMA}\right)}_{47}}$	Induction coefficient for GCLM gene	zmol^−1^.sec^−1^	LN (1.6, 3)	3.28
				5.75 ± 3.15
$k_{\mathrm{ind}{\left(\mathrm{NMA}\right)}_{56}}$	Induction coefficient for GST gene	zmol^−1^.sec^−1^	LN (11.9, 3)	8.46
				10.4 ± 8.61
$k_{\mathrm{ind}{\left(\mathrm{NMA}\right)}_{56b}}$	Induction coefficient for GPx gene	zmol^−1^.sec^−1^	LN (11.9, 3)	1.37
				6.75 ± 6.51
$k_{\mathrm{ind}{\left(\mathrm{NMA}\right)}_{65}}$	Induction coefficient for MRP gene	zmol^−1^.sec^−1^	LN (16, 3)	6.43
				9.62 ± 7.85
$v_{\max{\left(\mathrm{GCL}\right)}_{72}}$	Maximum rate of γ-GC synthesis by GCL	sec^−1^	LN (8.2, 3)	83.4
				80.3 ± 67.5
$v_{\max{\left(\mathrm{GCLC}\right)}_{72}}$	Maximum rate of γ-GC synthesis by GCLC	sec^−1^	LN (1.9, 3)	1.64
				2.16 ± 3.11
$v_{\mathrm{ma}x_{73}}$	Maximum rate of GSH synthesis	sec^−1^	LN (6.5, 3)	8.57
				10.3 ± 4.43
$v_{\mathrm{ma}x_{74}}$	Maximum rate of GSH degradation	zmol.sec^−1^	LN (1845, 3)	283
				374 ± 353
*Km*_74_	Michaelis-Menten constant of GSH degradation	zmol	LN (2 × 107, 3)	1.62 × 108
				2.22 × 108 ± 2.36 × 108

^*^: 1 zmol = 1 zeptomole = 10^−21^ mol.

^**^: *LN*: Log-normal distribution (mean, SD).

### Quantification of CsA toxicity for RPTECs

The CsA concentration causing a 50% decrease (EC50) in RPTECs’ viability was estimated from the data of Limonciel *et al.*[24] who report dose–response data on the viability of RPTECs, 3 T3 and HepaRG cells after exposure to various chemicals, including CsA. The dose range for CsA was not large enough to directly estimate an EC50, but a dose–response relationship could nevertheless be reconstructed by meta-analysis in a Bayesian framework, borrowing information from the full dose–response observed in the more sensitive 3 T3 and HepaRG cells. A standard decreasing log-logistic model [25] was calibrated to those dose-viability data using MCMC simulations (see Additional file 1: Section 3 for details).

### Software used

All model simulations and MCMC calibrations were performed with GNU MCSim v5.4.0 [4]. The R software, version 2.15.1 [26] was used for other statistical analyses and plots.

## Results

### *In vitro* pharmacokinetic model

**Figure 3:**
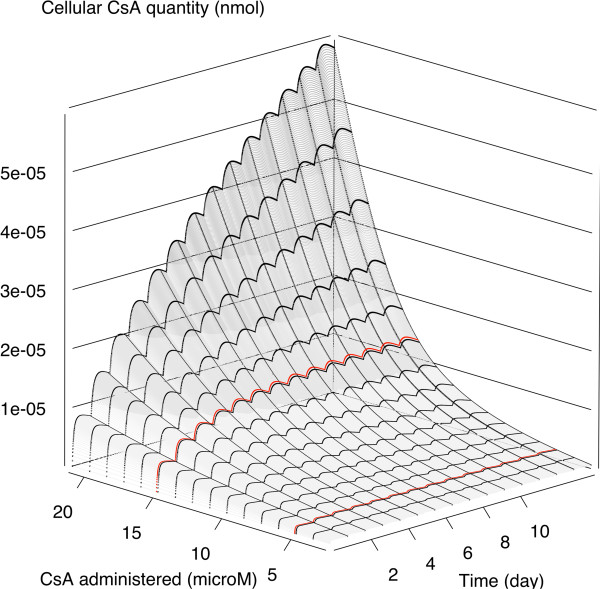
**Predictions of RPTECs intracellular CsA quantity versus time and dose during repeated dosing.** Thick red lines are predictions for 5 μM and 15 μM dosing.

Results for the *in vitro* pharmacokinetic model have been previously reported in Wilmes *et al.*[5] and are briefly summarized here. Overall, the data were well simulated (see Figure five in Wilmes *et al.*). Exposure to low concentrations of CsA (5 μM) led to a dynamic steady state in about 3 days. The average ratio between extracellular and intracellular CsA concentrations was about 200, consistent with the fact that CsA is lipophilic and accumulates in cells. Exposure to high concentrations of CsA (15 μM) led to major alterations of the biodistribution of CsA over time. Steady state was reached in the cells only after approximately 7 days. The average ratio of intracellular to extracellular CsA concentrations was about 200 on the first day (like at low concentration) and around 650 on the last day. Those results highlight the presence of nonlinear phenomena in the distribution of CsA in RPTECs. Note here the importance of administering repeated doses: A unique administration would not have uncovered those phenomena. Figure 3 show the influence of the extra-cellular concentration of CsA on the evolution of intra-cellular CsA quantities over time. Above about 15 μM extra-cellular CsA, intra-cellular concentration does not reach a plateau within 14 days.

### Coupled PK-systems biology model of the Nrf2 pathway

**Figure 4:**
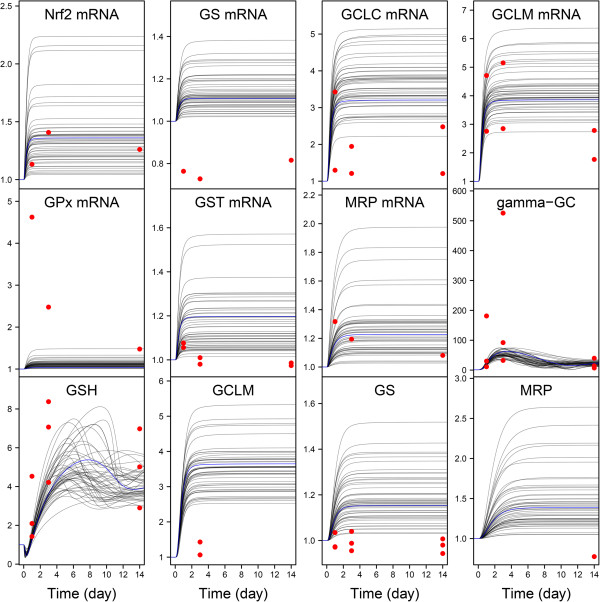
**Model fit to the omics data at low CsA exposure.** Transcriptomics (Nrf2 mRNA, GS mRNA, GCLC mRNA, GCLM mRNA, GST mRNA, GPx mRNA and ABCC2 mRNA) proteomics (GCLM, GS, and MRP2), and metabolomics (γ-GC, and GSH) fold-changes time-course in RPTEC cells during 14 days with repeated 5 μM CsA. The blue line indicates the best fitting (maximum posterior probability) model prediction. The black lines are predictions made with 49 parameter sets randomly drawn from their joint posterior distribution. The red circles represent data.

**Figure 5:**
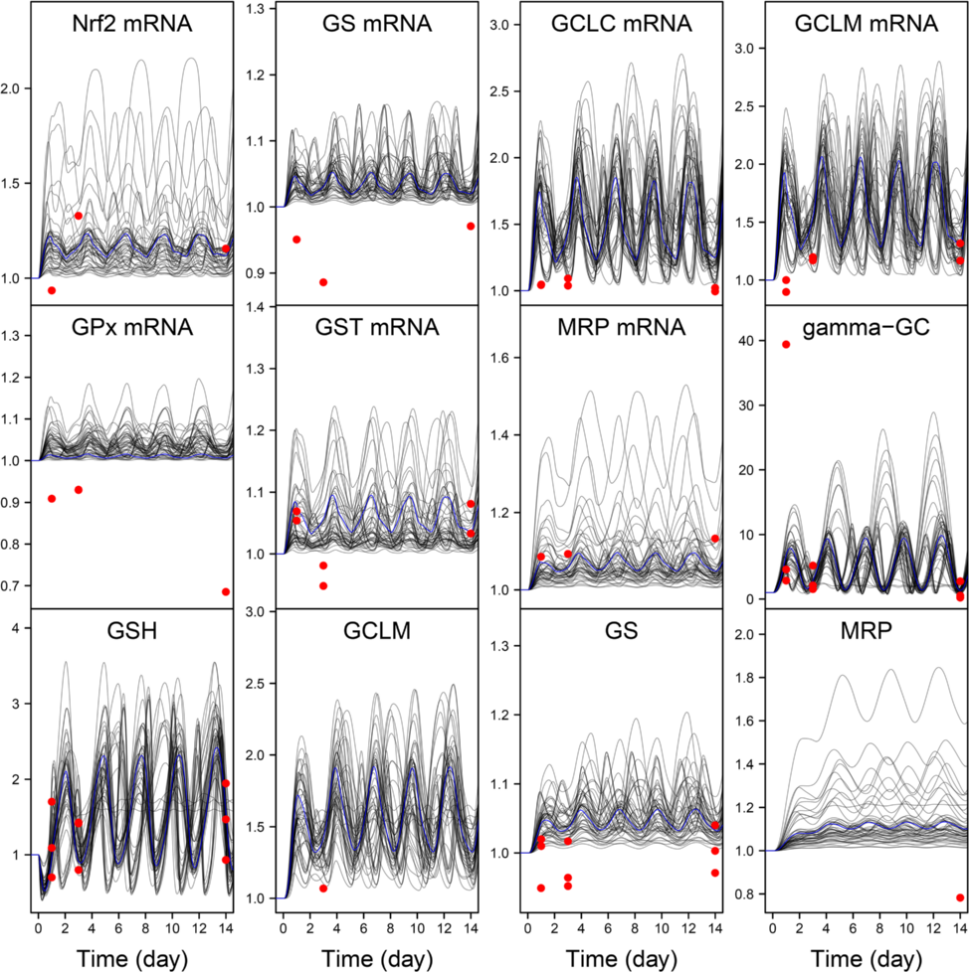
**Model fit to the omics data at high CsA exposure.** Transcriptomics (Nrf2 mRNA, GS mRNA, GCLC mRNA, GCLM mRNA, GST mRNA, GPx mRNA and ABCC2 mRNA) proteomics (GCLM, GS, and MRP2), and metabolomics (γ-GC, and GSH) fold-changes time-course in RPTEC cells during 14 days with repeated 15 μM CsA. The blue line indicates the best fitting (maximum posterior probability) model prediction. The black lines are predictions made with 49 parameter sets randomly drawn from their joint posterior distribution. The red circles represent data.

All the analyses and predictions presented here were made using a (posterior) random sample of 5000 parameter vectors, obtained by keeping one in each 100 of the second half of 200,000 iterations of five MCMC chains. Approximate convergence required that number of runs, and the median of the Gelman and Rubin’s criterion was 1.045. Figures 4 and 5 show the model fit obtained for the *in vitro* omics data, at low and high CsA exposure dose, respectively. The bundle of curves presented was obtained using the maximum posterior parameter vector and 49 other parameter sets randomly drawn from their joint posterior distribution. It reflects residual uncertainty in the model predictions, resulting from unavoidable measurement errors and modeling approximations. Overall the data (a total of 227 omics data values) are quite well fitted. After calibration, the relative differences between data and predictions are 38% on average (Additional file 1: Figure S3 shows an overall data *vs.* prediction plot for both *in vitro* PK and PD). That is somewhat above, but not by much, the expected measurement precision of the data. The worst fits are seen for the genes whose transcription apparently decreased, while the model could only predict an increased transcription. Yet, in most cases the decrease observed was modest (down from 1 to 0.7 at most). Also, the early and rather large increase in glutathione peroxidase mRNA at 5 μM CsA, with a decrease at 15 μM, could not be reproduced.

For all species, the time profiles are clearly different between the two doses. At 5 μM CsA exposure (Figure 4) periodic oscillations are pervasive. For many curves (including the most probable one) the system does not appear to have reached a dynamic equilibrium within 14 days. The oscillations’ period may differ from one curve to another and goes up to four days, even though the period of CsA administration was exactly one day. Additional file 1: Figure S4 extends the simulation length to 60 days, time at which a dynamic equilibrium is reached in all cases with the same oscillation pattern. At 15 μM CsA exposure (Figure 3) two patterns emerge: The first type of profile, which concerns all species except GSH and γ-GC, is a plateauing curve. Different maximum values are reached after three days by different curves. The second type, for GSH and γ-GC, starts with oscillations which do not stabilized within 14 days. Additional file 1: Figure S5 extends the simulation length to 60 days and shows more clearly the long-term behavior of GSH and γ-GC. The initial oscillations decrease gradually in amplitude and completely disappear after about 30 days.

**Figure 6:**
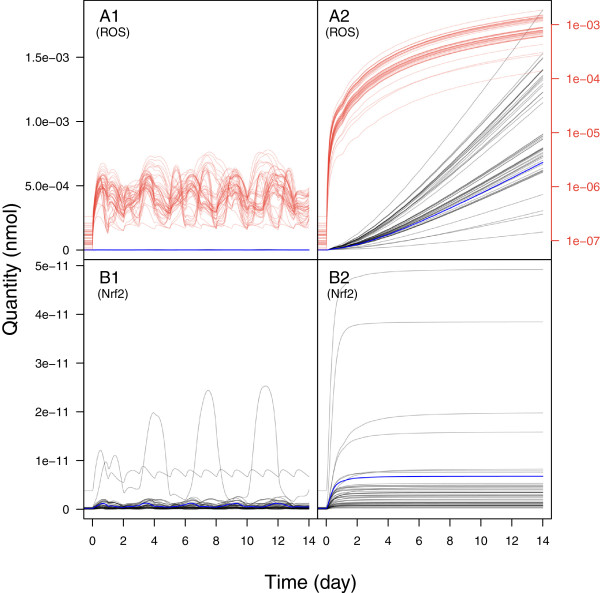
**Model predictions of the time course of ROS and Nrf2 protein after repeated CsA exposures.** Cytosolic ROS quantity after 5 μM 14 days repeated CsA exposure (A1), 15 μM CsA (A2) and nuclear Nrf2 quantity after 5 μM CsA exposure (B1) and 15 μM CsA (B2). The blue line indicates the best fitting (maximum posterior probability) model prediction. The black lines (normal scales) and red lines (semi-logarithmic scales) are predictions made with 49 random posterior parameter sets.

Figure 6 shows model predictions of the time course for the quantity of two non-observed chemical species – the free nuclear Nrf2 protein and cellular ROS – over 14 days, at either low or high repeated CsA dosing. Additional simulations were performed up to 60 days and the trends were similar (data not shown). As for the previous chemical species for which we had data, large differences are seen between low and high dosing. At low CsA exposure a cyclic pattern is observed, which disappears at high exposure where the ROS quantity grows (less than exponentially) while the Nrf2 protein quantity systematically reaches a plateau.

**Figure 7:**
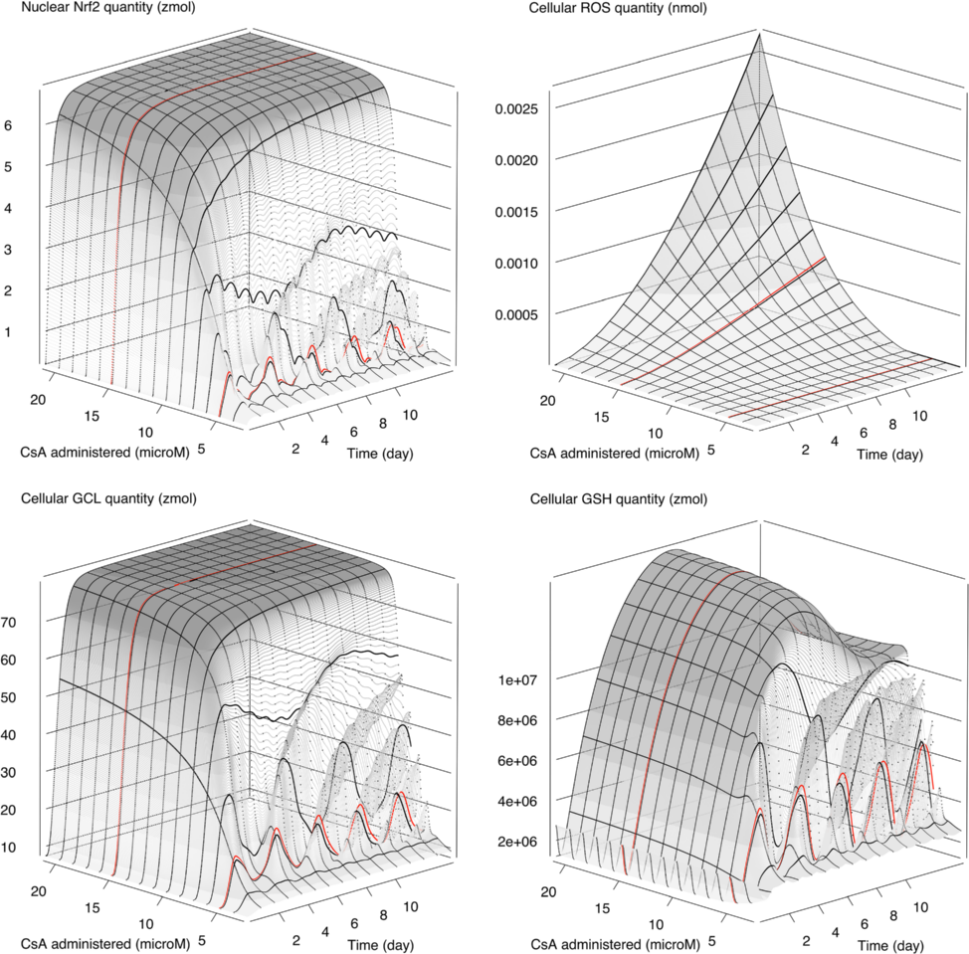
**Model predictions *vs. *time and CsA dose.** Predictions are shown for cellular ROS quantity (nmol) (top left), nuclear Nrf2 quantity (zmol) (top right), cellular GSH quantity (zmol) (down left) and cellular GCL quantity (zmol) (down right) quantities. The thick red lines are predictions for 5 μM and 15 μM CsA exposures.

Figure 7 shows 3D plots of the influence of the extracellular CsA concentration on the time course of cellular ROS, nuclear Nrf2 protein, cellular GSH and cellular GCL quantities. Figures for other species (GCLC, GCLM, GPx, GS and GST) are not shown because their profiles are very similar to the GCL one. The extracellular concentration of CsA has a large influence on the amount of ROS in the cytosol. For extracellular CsA concentrations below 8 μM CsA, the concentration (or quantity) *vs.* time profile of cytosolic ROS is oscillating, above 8 μM CsA, the ROS profile rises in a hockey-stick fashion. For nuclear Nrf2, cellular GSH and cellular GCL, depending on the extracellular CsA concentration, the model predicts either oscillating or plateauing profiles. As for ROS, the transition is rather abrupt and occurs approximately at 8 μM CsA.

### Comparison to CsA EC_50_ in RPTECs

Based on our laboratory data, an estimate of 38.5 μM (95% confidence interval: 24.5 μM to 69 μM) was obtained for the EC_50_ of CsA for its effect on RPTECs viability (details in Additional file 1: Section 1), after 14 days of repeated dosing. Note that the maximum concentration for CsA in water is about 50 μM so that value would actually be difficult to exceed. In any case, the above range of CsA EC_50_ in RPTECs matches the predicted increase in ROS beyond 15 μM seen on Figure 7.

## Discussion

A proper assessment of drug or chemical safety from *in vitro* assays requires the measurement of concentration of the parent molecule (and eventually its metabolites) in the assay medium and in cells [27,28]. Joint kinetic and effect modeling can then be used to interpolate and extrapolate the data obtained. Here, an *in vitro* pharmacokinetic model was first built using LC-MS/MS data on the distribution of CsA over time in human RPTECs. It was then extended to include a description of the Nrf2 pathway response to the resulting oxidative stress. CsA is highly lipophilic and its rapid uptake and accumulation in cells was observed. At 5 μM CsA (daily initial extracellular concentration), the model indicated that steady-state was reached in about 2 days, whereas at 15 μM CsA, steady-state was reached only after 7 days. Moreover, cellular CsA concentrations at steady-state were clearly not proportional to exposure, and a disproportionate accumulation of CsA was observed at high exposure. That could be explained by an interplay between the saturation of CsA metabolism and transport by P-glycoprotein out of the cells. However, the amount of CsA metabolized in our *in vitro* PK experiments on RPTECs seems limited to only about 15% of the total dose applied, at either low or high dose. So metabolism alone cannot explain the large increase in concentration that was observed. Also, if CsA entry and exit from RPTECs were simply linear, the ratio of intra-cellular to extra-cellular concentrations would stay constant in time, and both curves would be parallel on the log-scale (even while increasing because of metabolism saturation). However, the concentration ratio clearly increases with time and that is easily explained by the saturation of the P-glycoprotein efflux mechanism which was observed by Wilmes *et al.*[5] above 5 μM CsA exposure. Drug accumulation in target tissues is often associated with tissue-specific toxicities, and it is important to account for it. However, we did not observe a direct modulation of CsA PK by its PD in our *in vitro* system, even though CsA interactions with transporters are known [29]. In particular, CYP 3A5 levels were not affected by CsA levels, so CsA metabolism was not disturbed by induction or repression.

Zhang’s Nrf2 model was not intended to be used specifically with CsA or our cell system, so we had to re-calibrate several parameter values. This was done in a Bayesian statistical framework [21], to take into account the prior information we had on several parameters. Convergence of the MCMC simulations was difficult to obtain due to the high nonlinearity of the model and the presence of cycles. Basically almost any sub-multiple of the actual period would gives an acceptable fit, given the measurement noise. Fortunately, the prior parameter values documented by Zhang *et al.* stabilize the estimation process. It would be probably impossible to calibrate the model with a simple maximum likelihood approach (*i.e.*, without taking prior information into account). For most parameters, the *posterior* mean estimate was clearly different from the *prior* mean. The parameters controlling ROS formation (*kf*_75_) and ROS elimination (*v*_max8b_) had respectively higher and lower values, compared to Zhang’s model, after MCMC sampling. We centered our GPx parameters’ priors on the values used by Zhang *et al.* for GST. Since the observed GST and GPx transcriptomic data profiles were very different, it is not surprising that the posterior distributions of GPx parameters are clearly different from their prior. Via the Nrf2 pathway, CsA seems to have a large influence on glutamate cysteine ligase synthesis. While the basal transcription rates of GCLC and GCLM have posterior values close to those of Zhang’s *et al.*, parameters of GCLC and GCLM genes regulation by Nrf2, linked to ROS and CsA levels, have at values about four times higher. Indeed the model is imperfect in that it does not describe many additional controls and cross-talks with other pathways, or makes approximations. For example, GPx is specific for H_2_O_2_, and ROS are eliminated by a number of enzymes, some of which Nrf2 influences negatively. Consequently, for example, our model cannot explain the (small) decreases that were observed for some gene transcripts and does not describe well the time course of GPx mRNA at low dose. More observations or model refinements would be needed to understand the origin (noise in the data or an inappropriate model assumption) of that discrepancy.

The model still gives access to unmeasured effects of CsA to cells, closer to a toxicity endpoint. The generation of ROS by CsA is an important toxicity mechanism for that molecule. The retro-control of ROS scavenging by the Nrf2 signaling pathway induces a highly nonlinear behavior illustrated on Figure 7. The cyclic patterns observed at low CsA exposure (Figure 4 and Additional file 1: Figure S4) are interesting. A recent paper describes circadian oscillations of the Nrf2/GSH pathway in mice lung [30]. That pathway is well conserved and present in most cells since it regulates oxidative species generated during respiration. Therefore, a rhythmic pattern of Nrf2 activity in RPTECs would not be surprising even in the absence of CsA. In addition, the daily exposure of RPTECs to 5 μM CsA is likely to have produced a manageable burst of oxidative stress at the beginning of each day. That alone could explain the cycles seen, even if the nonlinear dynamics of the model result in a period of two to three days for those cycles. ROS generation runs out of control at CsA exposure levels close to the high dose assayed *in vitro* (15 μM for extra-cellular concentration) and the cycles disappear (Figure 5 and Additional file 1: Figure S5). We have an external corroboration of this finding: The 15 μM concentration was experimentally chosen to be the highest not affecting cell survival. Above that level, toxicity starts to have an impact on survival and the *in vitro* EC50 of CsA in RPTECS is probably close to 40 μM, so our model predictions seem reasonable. However, as in many systems biology models, only one signaling pathway has been taken into account. We also do not have extensive data allowing for an in-depth statistical cross-validation of the many components of the model. Other ROS scavenging mechanisms are present in RPTECs and could be involved during CsA exposure. On the other hand, CsA nephrotoxicity involves several mechanism [31-35] and it is possible that ROS generation is not alone in causing critical damages.

## Conclusion

Integrating omics approaches with mathematical systems biology models is still rarely done [36,37], even though that seems the best way to both understand the data and improve the predictive ability of the models [38,39]. Our modeling and simulations of the CsA mediated ROS production gives biologists insight into mechanisms of toxicity and provide quantitative estimates of toxicity beyond the time and dose range used in experiments. To go further, it would be interesting to have a more precise model description of GSH synthesis in the model, since cellular ROS concentrations are clearly correlated to GSH. It would also be interesting to couple this model with a physiological based pharmacokinetic (PBPK) model for CsA to be able to better predict human response. Still, our results demonstrate the possibility to use different omic data streams to extrapolate in time and dose the response of the Nrf2 pathway to oxidative damage, far beyond our current experimental possibilities.

## Abbreviations

ABCC2: ATP-binding cassette sub-family C member 2; AhR: Aryl hydrocarbon receptor (transcription factor protein); ARE: Antioxidant response element; ARNT: Aryl hydrocarbon receptor nuclear translocator (protein); CsA: Cyclosporine A; CsAcytosol: Cystosol CsA quantity; CsAextracellular: Extracellular CsA quantity; CsAwall: CsA on wall quantity; CYP: Cytochrome P450 3A5; DRE: Dioxin response element; GCL: Glutamate cysteine ligase; GCLC: Glutamate cysteine ligase catalytic subunit; GCLM: Glutamate cysteine ligase modifier subunit; GPx: Glutathione peroxidase; GS: Glutathione synthetase; GSH: Glutathione; GSSG: Glutathione disulfide; Keap1: Kelch-like ECH-associated protein 1; MCMC: Markov chain Monte Carlo; mRNA: Messenger ribonucleic acid; MRP2: Multidrug resistance associated protein 2; MTX: Metabolomic; NMA: Nrf2-Maf-ARE complex; Nrf2: Nuclear factor (erythroid-derived 2)-like 2; NRS: Non reactive species; PK: Pharmacokinetics; PTX: Proteomic; ROS: Reactive oxygen species; RPTEC: Renal proximal tubular epithelial cells; TCX: Transcriptomic; XAA: Complex of CsA-AhR-ARNT; XAAD: Complex of CsA-AhR-ARNT-DRE; γ-GC: γ-glutamylcysteine.

## Competing interests

The authors declare that they have no competing interests.

## Authors’ contributions

JH and FYB coded and ran the model. PJ performed the experiments. All authors participated in the redaction of this article. All authors read and approved the final manuscript.

## Supplementary Material

Additional file 1 **Section 1.** Differential equations of the Nrf2 pathway model’. **Section 2.** Preliminary sensitivity analysis for the selection of Nrf2 model parameters to calibrate. **Section 3.** Quantification of CsA toxicity for RPTECs. **Figure S1.** Maximum posterior fits of the log-logistic viability model for 3T3 and HepaRG cells viability data as a function of CsA exposure concentration. **Figure S2.** Maximum posterior fit and 95% confidence bounds of the log-logistic viability model for RPTECs viability data as a function of CsA exposure concentration. **Table S1.** Model parameters values and initial state variables values. **Table S2.** Cyclosporine A quantities measured in the extracellular medium at low CsA concentration exposure. **Table S3.** Intracellular Cyclosporine A quantities measured at low CsA concentration exposure. **Table S4.** Cyclosporine A quantities measured on plastic at low CsA concentration exposure. **Table S5.** Cyclosporine A quantities measured in the extracellular medium at high CsA concentration exposure. **Table S6.** Intracellular Cyclosporine A quantities measured at high CsA concentration exposure. **Table S7.** Cyclosporine A quantities measured on plastic at high CsA concentration exposure. **Table S8.** Fold changes measured at low CsA concentration. **Table S9.** Fold changes measured at high CsA concentration. **Figure S3.** Model fit to the data. The data values are plotted against the model predictions, after model calibration. The PK data are represented by black circles, the metabolomic data by green square, transcriptomic by red triangles and proteomics by blue inverted triangles. **Figure S4.** Transcriptomics, proteomics, and metabolomics (γ-GC, and GSH) fold-changes time-course in RPTEC cells during 60 days with repeated low dose CsA dosing. **Figure S5.** Transcriptomics, proteomics, and metabolomics (γ-GC, and GSH) fold-changes time-course in RPTEC cells during 60 days with repeated high dose CsA dosing. Click here for file
