# Supplementary material for: Systems biology modeling of omics data: effect of cyclosporine a on the Nrf2 pathway in human renal cells
Source: BMC Syst Biol. 2014 Jun 25;8:76. doi: 10.1186/1752-0509-8-76 (PMC4089556; doi:10.1186/1752-0509-8-76)
Supplement: Additional file 1 — Section 1. Differential equations of the Nrf2 pathway model’. Section 2. Preliminary sensitivity analysis for the selection of Nrf2 model parameters to calibrate. Section 3. Quantification of CsA toxicity for RPTECs. Figure S1. Maximum posterior fits of the log-logistic viability model for 3T3 and HepaRG cells viability data as a function of CsA exposure concentration. Figure S2. Maximum posterior fit and 95% confidence bounds of the log-logistic viability model for RPTECs viability data as a function of CsA exposure concentration. Table S1. Model parameters values and initial state variables values. Table S2. Cyclosporine A quantities measured in the extracellular medium at low CsA concentration exposure. Table S3. Intracellular Cyclosporine A quantities measured at low CsA concentration exposure. Table S4. Cyclosporine A quantities measured on plastic at low CsA concentration exposure. Table S5. Cyclosporine A quantities measured in the extracellular medium at high CsA concentration exposure. Table S6. Intracellular Cyclosporine A quantities measured at high CsA concentration exposure. Table S7. Cyclosporine A quantities measured on plastic at high CsA concentration exposure. Table S8. Fold changes measured at low CsA concentration. Table S9. Fold changes measured at high CsA concentration. Figure S3. Model fit to the data. The data values are plotted against the model predictions, after model calibration. The PK data are represented by black circles, the metabolomic data by green square, transcriptomic by red triangles and proteomics by blue inverted triangles. Figure S4. Transcriptomics, proteomics, and metabolomics (γ-GC, and GSH) fold-changes time-course in RPTEC cells during 60 days with repeated low dose CsA dosing. Figure S5. Transcriptomics, proteomics, and metabolomics (γ-GC, and GSH) fold-changes time-course in RPTEC cells during 60 days with repeated high dose CsA dosing. [file 1752-0509-8-76-S1.pdf]

SYSTEMS BIOLOGY MODELING OF OMICS DATA: EFFECT OF CYCLOSPORINE A ON THE NRF2 PATHWAY IN HUMAN RENAL KIDNEYS CELLS

Jérémy Hamon, Paul Jennings, Frédéric Y. Bois

ADDITIONAL MATERIAL

1. Differential equations of the Nrf2 pathway model:

$$\frac{\partial\, AhR_{cytosol}}{\partial t} = -k_{b_2} . CsA_{cytosol} . AhR_{cytosol} + k_{u_2} . CsA\_AhR_{cytosol} \quad (A1)$$

$$\frac{\partial\, AhR_{nucleus}}{\partial t} = -k_{b_5} . CsA_{nucleus} . AhR_{nucleus} + k_{u_5} . CsA\_AhR_{nucleus} \quad (A2)$$

$$\frac{\partial\, ARE_{GCLC}}{\partial t} = -k_{b_{40}} . ARE_{GCLC} \left(Nrf2\_MAF_{nucleus}\right)^{n_{40}} + k_{u_{40}} . NMA_{GCLC} \quad (A3)$$

$$\frac{\partial\, ARE_{GCLM}}{\partial t} = -k_{b_{46}} . ARE_{GCLM} \left(Nrf2\_MAF_{nucleus}\right)^{n_{46}} + k_{u_{46}} . NMA_{GCLM} \quad (A4)$$

$$\frac{\partial\, ARE_{GS}}{\partial t} = -k_{b_{32}} . ARE_{GS} \left(Nrf2\_MAF_{nucleus}\right)^{n_{32}} + k_{u_{32}} . NMA_{GS} \quad (A5)$$

$$\frac{\partial\, ARE_{GST}}{\partial t} \quad -k_{b_{54}} . ARE_{GST} \left(Nrf2\_MAF_{nucleus}\right)^{n_{54}} + k_{u_{54}} . NMA_{GST} \quad (A6)$$

$$\frac{\partial\, ARE_{GPx}}{\partial t} \quad -k_{b_{54b}} . ARE_{GPx} \left(Nrf2\_MAF_{nucleus}\right)^{n_{54b}} + k_{u_{54b}} . NMA_{GPx} \quad (A7)$$

$$\frac{\partial\, ARE_{MRP}}{\partial t} = -k_{b_{63}} . ARE_{MRP} \left(Nrf2\_MAF_{nucleus}\right)^{n_{63}} + k_{u_{63}} . NMA_{MRP} \quad (A8)$$

$$\frac{\partial\, ARE_{Nrf2}}{\partial t} = -k_{b_{25}} . ARE_{Nrf2} \left(Nrf2\_MAF_{nucleus}\right)^{n_{25}} + k_{u_{25}} . NMA_{Nrf2} \quad (A9)$$

$$\frac{\partial\, ARNT_{nucleus}}{\partial t} = -k_{b_6} . CsA\_AhR_{nucleus} . ARNT_{nucleus} + k_{u_6} . XAA_{nucleus} \quad (A10)$$

$$\frac{\partial CsA_{cytosol}}{\partial t} = CL_{in_1}.\frac{CsA_{extracellular}}{V_{extracellular}} - \frac{\frac{CL_{out_1}}{Km_{out_1}}.\frac{CsA_{cytosol}}{V_{cytosol}}}{1+\frac{CsA_{cytosol}}{V_{cytosol}.Km_{out_1}}} - \frac{v_{max_7}.CYP_{cytosol}.CsA_{cytosol}}{Km_2+CsA_{cytosol}} - k_{b_2}.CsA_{cytosol}.AhR_{cytosol}+k_{u_2}.CsA\_AhR_{cytosol} - CL_{in_4}.\frac{CsA_{cytosol}}{V_{cytosol}}+CL_{out_4}.\frac{CsA_{nucleus}}{V_{nucleus}} \quad \text{(A11)}$$

$$\frac{\partial CsA_{extracellular}}{\partial t} = -CL_{in_1}.\frac{CsA_{extracellular}}{V_{extracellular}} + \frac{\frac{CL_{out_1}}{Km_{out_1}}.\frac{CsA_{cytosol}}{V_{cytosol}}}{1+\frac{CsA_{cytosol}}{V_{cytosol}.Km_{out_1}}} - k_1.CsA_{extracellular}+k_2\left(CsA_{wall}\right)^{k_3} \quad \text{(A12)}$$

$$\frac{\partial CsA_{nucleus}}{\partial t} = -k_{b_5}.CsA_{nucleus}.AhR_{nucleus}+k_{u_5}.CsA\_AhR_{nucleus} + CL_{in_4}.\frac{CsA_{cytosol}}{V_{cytosol}} - CL_{out_4}.\frac{CsA_{nucleus}}{V_{nucleus}} \quad \text{(A13)}$$

$$\frac{\partial CsA_{wall}}{\partial t} = k_1.CsA_{extracellular}-k_2\left(CsA_{wall}\right)^{k_3} \quad \text{(A14)}$$

$$\frac{\partial CsA\_AhR_{cytosol}}{\partial t} = k_{b_2}.CsA_{cytosol}.AhR_{cytosol}-k_{u_2}.CsA\_AhR_{cytosol} - CL_{in_3}.\frac{CsA\_AhR_{cytosol}}{V_{cytosol}}+CL_{out_3}.\frac{CsA\_AhR_{nucleus}}{V_{nucleus}} \quad \text{(A15)}$$

$$\frac{\partial CsA\_AhR_{nucleus}}{\partial t} = CL_{in_3}.\frac{CsA\_AhR_{cytosol}}{V_{cytosol}}-CL_{out_3}.\frac{CsA\_AhR_{nucleus}}{V_{nucleus}} + k_{b_5}.CsA_{nucleus}.AhR_{nucleus}-k_{u_5}.CsA\_AhR_{nucleus} - k_{b_6}.CsA\_AhR_{nucleus}.ARNT_{nucleus}+k_{u_6}.XAA_{nucleus} \quad \text{(A16)}$$

$$\frac{\partial CYP_{cytosol}}{\partial t} = -k_{deg_{24}}.CYP_{cytosol}+k_{TSL_{23}}.mRNA_{CYP} \quad \text{(A17)}$$

$$\frac{\partial\, DRE_{CYP}}{\partial t} = -k_{b_{19}} . DRE_{CYP} \left(XAA_{nucleus}\right)^{n_{19}} + k_{u_{19}} . XAAD_{CYP} \quad (A18)$$

$$\frac{\partial\, DRE_{GST}}{\partial t} = -k_{b_{55}} . DRE_{GST} \left(XAA_{nucleus}\right)^{n_{55}} + k_{u_{55}} . XAAD_{GST} \quad (A19)$$

$$\frac{\partial\, DRE_{MRP}}{\partial t} = -k_{b_{64}} . DRE_{MRP} \left(XAA_{nucleus}\right)^{n_{64}} + k_{u_{64}} . XAAD_{MRP} \quad (A20)$$

$$\frac{\partial\, DRE_{Nrf2}}{\partial t} = -k_{b_{26}} . DRE_{Nrf2} \left(XAA_{nucleus}\right)^{n_{26}} + k_{u_{26}} . XAAD_{Nrf2} \quad (A21)$$

$$\frac{\partial\, GCL_{cytosol}}{\partial t} = k_{b_{52}} . GCLC_{cytosol} . GCLM_{cytosol} - k_{u_{52}} . GCL_{cytosol} - k_{deg_{53}} . GCL_{cytosol} \quad (A22)$$

$$\frac{\partial\, GCLC_{cytosol}}{\partial t} = -k_{b_{52}} . GCLC_{cytosol} . GCLM_{cytosol} + k_{u_{52}} . GCL_{cytosol} - k_{deg_{45}} . GCLC_{cytosol} + k_{TSL_{44}} . mRNA_{GCLC} \quad (A23)$$

$$\frac{\partial\, GCLM_{cytosol}}{\partial t} = -k_{b_{52}} . GCLC_{cytosol} . GCLM_{cytosol} + k_{u_{52}} . GCL_{cytosol} - k_{deg_{51}} . GCLM_{cytosol} + k_{TSL_{50}} . mRNA_{GCLM} \quad (A24)$$

$$\frac{\partial\, Gene_{CYP_{OFF}}}{\partial t} = -k_{act_{20}} . Gene_{CYP_{OFF}} - k_{ind_{20}} . Gene_{CYP_{OFF}} . XAAD_{CYP} + k_{desact_{20}} . Gene_{CYP_{ON}} \quad (A25)$$

$$\frac{\partial\, Gene_{CYP_{ON}}}{\partial t} = k_{act_{20}} . Gene_{CYP_{OFF}} + k_{ind_{20}} . Gene_{CYP_{OFF}} . XAAD_{CYP} - k_{desact_{20}} . Gene_{CYP_{ON}} \quad (A25)$$

$$\frac{\partial\, Gene_{GCLC_{OFF}}}{\partial t} = -k_{act_{41}} . Gene_{GCLC_{OFF}} - k_{ind_{41}} . Gene_{GCLC_{OFF}} . NMA_{GCLC} + k_{desact_{41}} . Gene_{GCCL_{ON}} \quad (A27)$$

$$\frac{Gene_{GCLC_{ON}}}{\partial t} = k_{act_{41}} . Gene_{GCLC_{OFF}} + k_{ind_{41}} . Gene_{GCLC_{OFF}} . NMA_{GCLC} - k_{desact_{41}} . Gene_{GCLC_{ON}} \quad (A28)$$

$$\frac{\partial\, Gene_{GCLM_{OFF}}}{\partial\, t} = -k_{act_{47}}.Gene_{GCLM_{OFF}} - k_{ind_{47}}.Gene_{GCLM_{OFF}}.NMA_{GCLM} + k_{desact_{47}}.Gene_{GCLC_{ON}} \quad \text{(A29)}$$

$$\frac{\partial\, Gene_{GCLM_{ON}}}{\partial\, t} = k_{act_{47}}.Gene_{GCLM_{OFF}} + k_{ind_{47}}.Gene_{GCLM_{OFF}}.NMA_{GCLM} - k_{desact_{47}}.Gene_{GCLM_{ON}} \quad \text{(A30)}$$

$$\frac{\partial\, Gene_{GS_{OFF}}}{\partial\, t} = -k_{act_{33}}.Gene_{GS_{OFF}} - k_{ind_{33}}.Gene_{GS_{OFF}}.NMA_{GS} + k_{desact_{33}}.Gene_{GS_{ON}} \quad \text{(A31)}$$

$$\frac{\partial\, Gene_{GS_{ON}}}{\partial\, t} = k_{act_{33}}.Gene_{GS_{OFF}} + k_{ind_{33}}.Gene_{GS_{OFF}}.NMA_{GS} - k_{desact_{33}}.Gene_{GS_{ON}} \quad \text{(A32)}$$

$$\frac{Gene_{GST_{OFF}}}{\partial\, t} = -k_{act_{56}}.Gene_{GST_{OFF}} - k_{ind(NMA)_{56}}.Gene_{GST_{OFF}}.NMA_{GST} - k_{ind(XAAD)_{56}}.Gene_{GST_{OFF}}.XAAD_{GST} + k_{desact_{56}}.Gene_{GST_{ON}} \quad \text{(A33)}$$

$$\frac{Gene_{GST_{ON}}}{\partial\, t} = k_{act_{56}}.Gene_{GST_{OFF}} + k_{ind(NMA)_{56}}.Gene_{GST_{OFF}}.NMA_{GST} \; k_{ind(XAAD)_{56}}.Gene_{GST_{OFF}}.XAAD_{GST} - k_{desact_{56}}.Gene_{GST_{ON}} \quad \text{(A34)}$$

$$\frac{Gene_{GPx_{OFF}}}{\partial\, t} = -k_{act_{56b}}.Gene_{GPx_{OFF}} - k_{ind(NMA)_{56b}}.Gene_{GPx_{OFF}}.NMA_{GPx} + k_{desact_{56b}}.Gene_{GPx_{ON}} \quad \text{(A35)}$$

$$\frac{Gene_{GPx_{ON}}}{\partial\, t} = k_{act_{56b}}.Gene_{GPx_{OFF}} + k_{ind(NMA)_{56b}}.Gene_{GPx_{OFF}}.NMA_{GPx} - k_{desact_{56b}}.Gene_{GPx_{ON}} \quad \text{(A36)}$$

$$\frac{\partial\, Gene_{MRP_{OFF}}}{\partial\, t} = -k_{act_{65}}.Gene_{MRP_{OFF}} - k_{ind(NMA)_{65}}.Gene_{MRP_{OFF}}.NMA_{MRP} - k_{ind(XAAD)_{65}}.Gene_{MRP_{OFF}}.XAAD_{MRP} + k_{desact_{65}}.Gene_{MRP_{ON}} \quad \text{(A37)}$$

$$\frac{\partial Gene_{MRP_{ON}}}{\partial t} = k_{act_{65}}.Gene_{MRP_{OFF}} + k_{ind(NMA)_{65}}.Gene_{MRP_{OFF}}.NMA_{MRP} + k_{ind(XAAD)_{65}}.Gene_{MRP_{OFF}}.XAAD_{MRP} - k_{desact_{65}}.Gene_{MRP_{ON}} \quad \text{(A38)}$$

$$\frac{\partial Gene_{Nrf2_{OFF}}}{\partial t} = -k_{act_{27}}.Gene_{Nrf2_{OFF}} - k_{ind(NMA)_{27}}.Gene_{Nrf2_{OFF}}.NMA_{Nrf2} - k_{ind(XAAD)_{27}}.Gene_{Nrf2_{OFF}}.XAAD_{Nrf2} + k_{desact_{27}}.Gene_{Nrf2_{ON}} \quad \text{(A39)}$$

$$\frac{\partial Gene_{Nrf2_{ON}}}{\partial t} = k_{act_{27}}.Gene_{Nrf2_{OFF}} + k_{ind(NMA)_{27}}.Gene_{Nrf2_{OFF}}.NMA_{Nrf2} + k_{ind(XAAD)_{27}}.Gene_{Nrf2_{OFF}}.XAAD_{Nrf2} - k_{desact_{27}}.Gene_{Nrf2_{ON}} \quad \text{(A40)}$$

$$\frac{\partial GS_{cytosol}}{\partial t} = k_{b_{38}}.GS\,mono_{cytosol}.GS\,mono_{cytosol} - k_{u_{38}}.GS_{cytosol} - k_{deg_{39}}.GS_{cytosol} \quad \text{(A41)}$$

$$\frac{\partial GS\,mono_{cytosol}}{\partial t} = -2.k_{b_{38}}.GS\,mono_{cytosol}.GS\,mono_{cytosol} + 2.\mathrm{k}_{u_{38}}.GS_{cytosol} - k_{deg_{37}}.GS\,mono_{cytosol} + k_{TSL_{36}}.mRNA_{GS} \quad \text{(A42)}$$

$$R11 = \frac{ATP_{cytosol}}{K_{m(ATP)(GCL)_{72}}\left(1+\frac{GSH_{cytosol}}{K_{is(ATP)(GCL)_{72}}}\right) + ATP_{cytosol}\left(1+\frac{GSH_{cytosol}}{K_{ii(ATP)(GCL)_{72}}}\right)} \quad \text{(A43)}$$

$$R12 = \frac{Glu_{cytosol}}{K_{m(Glu)(GCL)_{72}}\left(1+\frac{GSH_{cytosol}}{K_{is(Glu)(GCL)_{72}}}\right) + Glu_{cytosol}\left(1+\frac{GSH_{cytosol}}{K_{ii(Glu)(GCL)_{72}}}\right)} \quad \text{(A44)}$$

$$R13 = \frac{v_{max(GCL)_{72}}.GCL_{cytosol}.Cys_{cytosol}}{K_{m(Cys)(GCL)_{72}} + Cys_{cytosol}} \quad \text{(A45)}$$

$$R21 = \frac{ATP_{cytosol}}{K_{m(ATP)(GCLC)_{72}}\left(1+\frac{GSH_{cytosol}}{K_{is(ATP)(GCLC)_{72}}}\right) + ATP_{cytosol}\left(1+\frac{GSH_{cytosol}}{K_{ii(ATP)(GCLC)_{72}}}\right)} \quad \text{(A46)}$$

$$R22 = \frac{Glu_{cytosol}}{K_{m(Glu)(GCLC)_{72}}\left(1+\frac{GSH_{cytosol}}{K_{is(Glu)(GCLC)_{72}}}\right)+Glu_{cytosol}\left(1+\frac{GSH_{cytosol}}{K_{ii(Glu)(GCLC)_{72}}}\right)} \quad \text{(A46)}$$

$$R23 = \frac{v_{max(GCLC)_{72}}.GCLC_{cytosol}.Cys_{cytosol}}{K_{m(Cys)(GCLC)_{72}}+Cys_{cytosol}} \quad \text{(A48)}$$

$$R31 = \frac{\frac{\gamma GC_{cytosol}}{K_{m1(\gamma GC)_{73}}}+\frac{\gamma GC^2_{cytosol}}{K_{m1(\gamma GC)_{73}}.K_{m2(\gamma GC)_{73}}}}{1+\frac{2.\gamma GC_{cytosol}}{K_{m1(\gamma GC)_{73}}}+\frac{\gamma GC^2_{cytosol}}{K_{m1(\gamma GC)_{73}}.K_{m2(\gamma GC)_{73}}}} \quad \text{(A49)}$$

$$R32 = \frac{v_{max_{73}}.GS_{cytosol}.Gly_{cytosol}.ATP_{cytosol}}{\left(K_{m(Gly)_{73}}+Gly_{cytosol}\right)\left(K_{m(ATP)_{73}}+ATP_{cytosol}\right)} \quad \text{(A50)}$$

$$\frac{\partial \gamma GC_{cytosol}}{\partial t} = R11\times R12\times R13+R21\times R22\times R23-R31\times R32 \quad \text{(A51)}$$

$$\frac{\partial GSH_{cytosol}}{\partial t} = -\frac{v_{max_{74}}.GSH_{cytosol}}{K_{m_{74}}+GSH_{cytosol}}+R31\times R32 - \frac{v_{max_{8b}}.GPx_{cytosol}.GSH_{cytosol}}{K_{m(GSH)_{8b}}+GSH_{cytosol}}.\frac{ROS_{cytosol}}{K_{m(ROS)_{8b}}+ROS_{cytosol}} \quad \text{(A52)}$$

$$\frac{\partial GST_{cytosol}}{\partial t} = k_{b_{61}}.GST\,mono_{cytosol}.GST\,mono_{cytosol}-k_{u_{61}}.GST_{cytosol} - k_{deg_{62}}.GST_{cytosol} \quad \text{(A53)}$$

$$\frac{\partial GST\,mono_{cytosol}}{\partial t} = -2.k_{b_{61}}.GST\,mono_{cytosol}.GST\,mono_{cytosol}+2.k_{u_{61}}.GST_{cytosol} - k_{deg_{60}}.GST\,mono_{cytosol}+k_{TSL_{59}}.mRNA_{GST} \quad \text{(A54)}$$

$$\frac{\partial Keap1_{cytosol}}{\partial t} = k_{red_{10}}.Keap1o_{cytosol}-k_{ox_{10}}.Keap1_{cytosol}.ROS_{cytosol} + k_{u_{14}}.Nrf2\_Keap1_{cytosol}-k_{b_{14}}.Nrf2_{cytosol}.Keap1_{cytosol} + k_{u_{12}}.Nrf2\_Keap1_{cytosol} \quad \text{(A55)}$$

$$\frac{\partial\, Keap1o_{cytosol}}{\partial t} = -k_{red_{10}}.Keap1o_{cytosol}+k_{ox_{10}}.Keap1_{cytosol}.ROS_{cytosol} + k_{u_{15}}.Nrf2\_Keap1o_{cytosol}-k_{b_{15}}.Nrf2_{cytosol}.Keap1o_{cytosol} + k_{u_{13}}.Nrf2\_Keap1o_{cytosol} \quad (A56)$$

$$\frac{\partial\, mRNA_{CYP}}{\partial t} = -k_{deg_{22}}.mRNA_{CYP}+k_{TSP_{21}}.Gene_{CYP_{ON}} \quad (A57)$$

$$\frac{\partial\, mRNA_{GCLC}}{\partial t} = -k_{deg_{43}}.mRNA_{GCLC}+k_{TSP_{42}}.Gene_{GCLC_{ON}} \quad (A58)$$

$$\frac{\partial\, mRNA_{GCLM}}{\partial t} = -k_{deg_{49}}.mRNA_{GCLM}+k_{TSP_{48}}.Gene_{GCLM_{ON}} \quad (A59)$$

$$\frac{\partial\, mRNA_{GS}}{\partial t} = -k_{deg_{35}}.mRNA_{GS}+k_{TSP_{34}}.Gene_{GS_{ON}} \quad (A60)$$

$$\frac{\partial\, mRNA_{GST}}{\partial t} = -k_{deg_{58}}.mRNA_{GST}+k_{TSP_{57}}.Gene_{GST_{ON}} \quad (A61)$$

$$\frac{\partial\, mRNA_{GPx}}{\partial t} = -k_{deg_{58b}}.mRNA_{GPx}+k_{TSP_{57b}}.Gene_{GPx_{ON}} \quad (A62)$$

$$\frac{\partial\, mRNA_{MRP}}{\partial t} = -k_{deg_{67}}.mRNA_{MRP}+k_{TSP_{66}}.Gene_{MRP_{ON}} \quad (A63)$$

$$\frac{\partial\, mRNA_{Nrf2}}{\partial t} = -k_{deg_{29}}.mRNA_{Nrf2}+k_{TSP_{28}}.Gene_{Nrf2_{ON}} \quad (A64)$$

$$\frac{\partial\, MRP_{cytosol}}{\partial t} = k_{b_{70}}.MRP\,mono_{cytosol}.MRP\,mono_{cytosol}-k_{u_{70}}.MRP_{cytosol} -k_{deg_{71}}.MRP_{cytosol} \quad (A65)$$

$$\frac{\partial\, MRP\,mono_{cytosol}}{\partial t} = -2.k_{b_{70}}.MRP\,mono_{cytosol}.MRP\,mono_{cytosol}+2.\mathrm{k}_{u_{70}}.MRP_{cytosol} -k_{deg_{69}}.MRP\,mono_{cytosol}+k_{TSL_{68}}.mRNA_{MRP} \quad (A66)$$

$$\frac{\partial\, MAF_{nucleus}}{\partial t} = -k_{b_{18}}.MAF_{nucleus}.Nrf2_{nucleus}+k_{u_{18}}.Nrf2\_MAF_{nucleus} \quad (A67)$$

$$\frac{\partial NMA_{GCLC}}{\partial t} = k_{b_{40}} . ARE_{GCLC} \left(Nrf2\_MAF_{nucleus} \right)^{n_{40}} - k_{u_{40}} . NMA_{GCLC} \tag{A68}$$

$$\frac{\partial NMA_{GCLM}}{\partial t} = k_{b_{46}} . ARE_{GCLM} \left(Nrf2\_MAF_{nucleus} \right)^{n_{46}} - k_{u_{46}} . NMA_{GCLM} \tag{A69}$$

$$\frac{\partial NMA_{GS}}{\partial t} = k_{b_{32}} . ARE_{GS} \left(Nrf2\_MAF_{nucleus} \right)^{n_{32}} - k_{u_{32}} . NMA_{GS} \tag{A70}$$

$$\frac{\partial NMA_{GST}}{\partial t} = k_{b_{54}} . ARE_{GST} \left(Nrf2\_MAF_{nucleus} \right)^{n_{54}} - k_{u_{54}} . NMA_{GST} \tag{A71}$$

$$\frac{\partial NMA_{GPx}}{\partial t} = k_{b_{54b}} . ARE_{GPx} \left(Nrf2\_MAF_{nucleus} \right)^{n_{54b}} - k_{u_{54b}} . NMA_{GPx} \tag{A72}$$

$$\frac{\partial NMA_{MRP}}{\partial t} = k_{b_{63}} . ARE_{MRP} \left(Nrf2\_MAF_{nucleus} \right)^{n_{63}} - k_{u_{63}} . NMA_{MRP} \tag{A73}$$

$$\frac{\partial NMA_{Nrf2}}{\partial t} = k_{b_{25}} . ARE_{Nrf2} \left(Nrf2\_MAF_{nucleus} \right)^{n_{25}} - k_{u_{25}} . NMA_{Nrf2} \tag{A74}$$

$$\begin{aligned} \frac{\partial Nrf2_{cytosol}}{\partial t} = & -k_{deg_{31}} . Nrf2_{cytosol} + k_{TSL_{30}} . mRNA_{Nrf2} \\ & -k_{b_{15}} . Nrf2_{cytosol} . Keap1o_{cytosol} + k_{u_{15}} . Nrf2\_Keap1o_{cytosol} \\ & -k_{b_{14}} . Nrf2_{cytosol} . Keap1_{cytosol} + k_{u_{14}} . Nrf2\_Keap1_{cytosol} \\ & -CL_{in_{16}} . \frac{Nrf2_{cytosol}}{V_{cytosol}} + CL_{out_{16}} . \frac{Nrf2_{nucleus}}{V_{nucleus}} \end{aligned} \tag{A75}$$

$$\begin{aligned} \frac{\partial Nrf2_{nucleus}}{\partial t} = & -k_{b_{18}} . Nrf2_{nucleus} . Maf_{nucleus} + k_{u_{18}} . Nrf2\_Maf_{nucleus} \\ & +CL_{in_{16}} . \frac{Nrf2_{cytosol}}{V_{cytosol}} - CL_{out_{16}} . \frac{Nrf2_{nucleus}}{V_{nucleus}} \\ & -k_{deg_{17}} . Nrf2_{nucleus} \end{aligned} \tag{A76}$$

$$\frac{\partial\, Nrf2\_Keap1_{cytosol}}{\partial t} = k_{red_{11}} . Nrf2\_Keap1o_{cytosol} - k_{ox_{11}} . Nrf2\_Keap1_{cytosol} . ROS_{cytosol} - k_{u_{14}} . Nrf2\_Keap1_{cytosol} + k_{b_{14}} . Nrf2_{cytosol} . Keap1_{cytosol} - k_{u_{12}} . Nrf2\_Keap1_{cytosol} \quad \text{(A77)}$$

$$\frac{\partial\, Nrf2\_Keap1o_{cytosol}}{\partial t} = -k_{red_{11}} . Nrf2\_Keap1o_{cytosol} + k_{ox_{11}} . Nrf2\_Keap1_{cytosol} . ROS_{cytosol} - k_{u_{15}} . Nrf2\_Keap1o_{cytosol} + k_{b_{15}} . Nrf2_{cytosol} . Keap1o_{cytosol} - k_{u_{13}} . Nrf2\_Keap1o_{cytosol} \quad \text{(A78)}$$

$$\begin{aligned}\frac{\partial\, Nrf2\_Maf_{nucleus}}{\partial t} = & -2.k_{b_{54}} . ARE_{GST}\left(Nrf2\_MAF_{nucleus}\right)^{n_{54}} + 2.k_{u_{54}} . NMA_{GST} \\ & -2.k_{b_{54b}} . ARE_{GPx}\left(Nrf2\_MAF_{nucleus}\right)^{n_{54b}} + 2.k_{u_{54b}} . NMA_{GPx} \\ & -3.k_{b_{46}} . ARE_{GCLM}\left(Nrf2\_MAF_{nucleus}\right)^{n_{46}} + 3.k_{u_{46}} . NMA_{GCLM} \\ & -k_{b_{25}} . ARE_{Nrf2}\left(Nrf2\_MAF_{nucleus}\right)^{n_{25}} + k_{u_{25}} . NMA_{Nrf2} \\ & -2.k_{b_{63}} . ARE_{MRP} \cdot \left(Nrf2\_MAF_{nucleus}\right)^{n_{63}} + 2.k_{u_{63}} . NMA_{MRP} \\ & -3.k_{b_{40}} . ARE_{GCLC}\left(Nrf2\_MAF_{nucleus}\right)^{n_{40}} + 3.k_{u_{40}} . NMA_{GCLC} \\ & -2.k_{b_{32}} . ARE_{GS}\left(Nrf2\_MAF_{nucleus}\right)^{n_{32}} + 2.k_{u_{32}} . NMA_{GS} \\ & +k_{b_{18}} . Nrf2_{nucleus} . MAF_{nucleus} - k_{u_{18}} . Nrf2\_MAF_{nuleus} \end{aligned} \quad \text{(A79)}$$

$$\frac{\partial\, ROS_{cytosol}}{\partial t} = k_{f_{75}} + k_{ROS} . CsA_{cytosol} - \frac{v_{max_{8b}} . GPx_{cytosol} . GSH_{cytosol}}{K_{m(GSH)_{8b}} + GSH_{cytosol}} . \frac{ROS_{cytosol}}{K_{m(ROS)_{8b}} + ROS_{cytosol}} \quad \text{(A80)}$$

$$
\begin{aligned}
\frac{\partial XAA_{nucleus}}{\partial t} = & -2.k_{b_{26}}.DRE_{Nrf2}\left(XAA_{nucleus}\right)^{n_{26}}+2.k_{u_{26}}.XAAD_{Nrf2} \\
& -2.k_{b_{19}}.DRE_{CYP}\left(XAA_{nucleus}\right)^{n_{19}}+2.k_{u_{19}}.XAAD_{CYP} \\
& -2.k_{b_{55}}.DRE_{GST}\left(XAA_{nucleus}\right)^{n_{55}}+2.k_{u_{55}}.XAAD_{GST} \\
& -k_{b_{64}}.DRE_{MRP}\left(XAA_{nucleus}\right)^{n_{64}}+k_{u_{64}}.XAAD_{MRP} \\
& +k_{b_6}.X\_AhR_{nucleus}.ARNT_{nucleus}-k_{u_6}.XAA_{nucleus}
\end{aligned} \quad \text{(A81)}
$$

$$\frac{\partial XAAD_{CYP}}{\partial t} = k_{b_{19}}.DRE_{CYP}\left(XAA_{nucleus}\right)^{n_{19}}-k_{u_{19}}.XAAD_{CYP} \quad \text{(A82)}$$

$$\frac{\partial XAAD_{GST}}{\partial t} = k_{b_{55}}.DRE_{GST}\left(XAA_{nucleus}\right)^{n_{55}}-k_{u_{55}}.XAAD_{GST} \quad \text{(A83)}$$

$$\frac{\partial XAAD_{MRP}}{\partial t} = k_{b_{64}}.DRE_{MRP}\left(XAA_{nucleus}\right)^{n_{64}}-k_{u_{64}}.XAAD_{MRP} \quad \text{(A84)}$$

$$\frac{\partial XAAD_{Nrf2}}{\partial t} = k_{b_{26}}.DRE_{Nrf2}\left(XAA_{nucleus}\right)^{n_{26}}-k_{u_{26}}.XAAD_{Nrf2} \quad \text{(A85)}$$

2. Preliminary sensitivity analysis for the selection of nrf2 model parameters to calibrate

The set of parameters to calibrate was chosen on the basis of our understanding of the model structure and a preliminary sensitivity analysis. All model parameters were assigned a log-normal prior around their prior mean (either from Zhang et al., 2009, or from our *in vitro* CsA PK analysis) with a geometric SD of 10. The 1st to 99th percentile range of such distributions cover about five orders of magnitude. The parameters were jointly sampled by Monte Carlo simulations (n = 1000) and all data values were simulated. The likelihood of the data was evaluated at each iteration (assuming log-normal errors and a geometric SD of 1.3, *i.e.*, about 30% relative error). The slope of the line joining the point of highest likelihood and the next highest was taken as a measure of local curvature near the maximum likelihood and therefore as a sensitivity coefficient (parameters for which the curvature is small have little influence on the likelihood and hence on the data, and would be poorly estimated from those data). The resulting sensitivity coefficients ranged from 0.11 to 11.

Most of the sensitive parameters had either a direct influence on ROS synthesis, metabolism and interaction with Keap1 (sensitivity coefficients between 1 and 5), or controled the activation and

induction of Nrf2, GCLC, GCLM, GST, GPx, CYP3A5, GS and MRP genes (sensitivites between 0.5 and 7), or the synthesis and degradation of γ-GC and GSH (sensitivites between 0.7 and 5.3). However, those results were based, by force, on the sensitivity of the uncalibrated model and therefore very approximative. We therefore complemented this analysis by our expert judgement on the key model components, and decided to calibrate:

- the parameters directly related to CsA interactions with the other model components (because the original model proposed by Zhang *et al.* was not at all specific of CsA),
- the most crucial ROS-nrf2 parameters,
- the transcription rate constants (k_{TSB}) and transcription induction coefficients (k_{ind}) for all the nrf2 controled genes,
- the maximal rates for the GSH and γ-GC reactions and the K_m for GSH degradation.

3. Quantification of CsA toxicity for RPTECs

We obtained in our lab data on RPTECs, 3T3 and HepaRG cells viability after 14 days repeated exposure to a set of extracellular CsA concentrations. This is part of a paper published by Limonciel *et al.* (2011, "Lactate is an ideal non-invasive marker for evaluating temporal alterations in cell stress and toxicity in repeat dose testing regimes." Toxicology in Vitro 25: 1855-1862). The detailed data are given in the following table:

CsA (µM)	Viability (% of control)							
	RPTEC (3 replicates)			3T3 (duplicates)		HepaRG (3 replicates)		
0.2	108.6	90.89	110.2	103.8	87.56	102.3	96.99	112.3
2	127.1	87.86	103.1	105.9	105.1	78.42	97.45	102.7
5	73.95	81.38	89.98	77.70	79.12	31.58	41.35	52.22
15	91.97	76.38	78.59	0.009	-0.3238	2.906	4.898	4.892
20	86.67	74.39	93.09	-0.2648	-0.3053	2.470	3.252	7.717

There is a clear decline in viability of RPTEC cells at high CsA exposure, but the EC_{50} is not directly estimable, as it is for 3T3 and HepaRG cells. The following dose-response model was used:

$$y = 100 \frac{EC_{50}^n}{EC_{50}^n + C^n}$$

where C is the CsA concentration (in μM), y the viability (in %). EC_{50} and the power n are the two parameters of this model.

Posterior distribution of those parameters for 3T3 and HepaRG cells were first obtained using uninformative log-uniform priors and a standard normal likelihood. A common n and two separate EC_{50} were estimated by MCMC simulations (200000 iterations, convergence confirmed by Gelman and Rubin's diagnostic). Their posterior mean and SD estimates were:

Parameter	Mean	SD	2.5%tile	97.5%tile
n	4.61	1.32	3.02	8.23
log(EC_{50}) for 3T3	1.9	0.0822	1.75	2.08
log(EC_{50}) for HepaRG	1.53	0.0449	1.43	1.61

The maximum posterior fits for the 3T3 and HepaRG data are given in the next Figure:

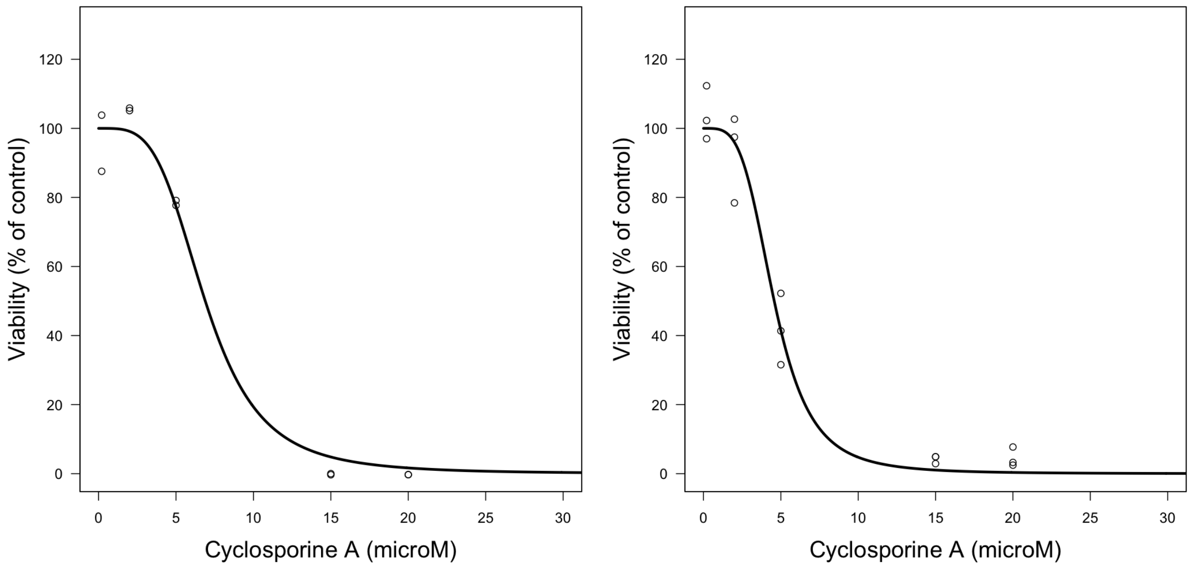

Figure S1: Maximum posterior fits (line) of the log-logistic viability model for 3T3 (left panel) and HepaRG cells (right panel) viability data (points) as a function of CsA exposure concentration. The estimated measurement error is about 7%.

The model was then calibrated in the same way with the RPTEC data, except that the above posterior mean and SD for n were used an informative prior (therefore borrowing information about n from the 3T3 and HepaRG data). The resulting posterior mean and SD for the RPTECs parameters were:

Parameter	Mean	SD	2.5%tile	97.5%tile
n	3.79	1.04	3.20	7.47
log(EC_{50}) for RPTEC	3.65	0.45	3.20	4.23

The maximum posterior fit obtained for RPTECs data, together with 95% confidence bounds, is shown in the next Figure.

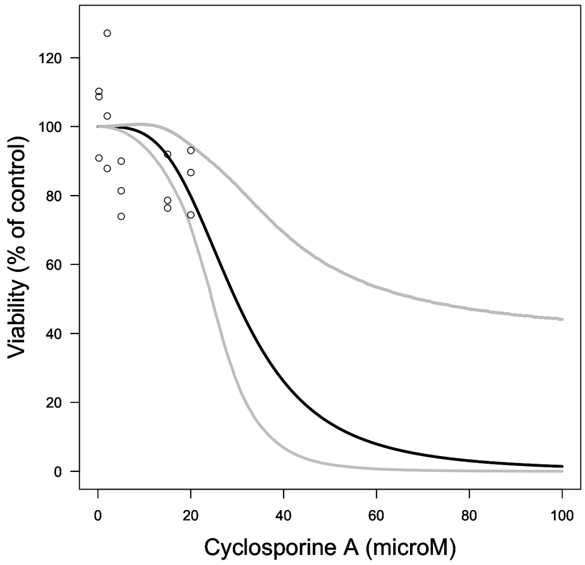

Figure S2: Maximum posterior fit (black line) and 95% confidence bounds (grey lines) of the log-logistic viability model for RPTECs viability data (points) as a function of CsA exposure concentration. The estimated measurement error is about 10%.

Table S1: Model parameters values and initial state variables values.

Equation	Parameters and initial state variables		
A1: $\frac{\partial AhR_{cytosol}}{\partial t}$	$AhR_{cytosol_{(initial)}}$ = 34 zmol	k_{b_2} = 0 $zmol^{-1}.s^{-1}$	k_{u_2} = 0.02 s^{-1}
A2: $\frac{\partial AhR_{nucleus}}{\partial t}$	$AhR_{nucleus_{(initial)}}$ = 0 zmol	k_{b_5} = 0 $zmol^{-1}.s^{-1}$	k_{u_5} = 0.02 s^{-1}
A3: $\frac{\partial ARE_{GCLC}}{\partial t}$	$k_{b_{40}}$ = 14.6 $zmol^{-3}.s^{-1}$	$k_{u_{40}}$ = 0.02 s^{-1}	n_{40} = 3
		$ARE_{GCLC_{(initial)}}$ = 6.35E^{-4} zmol	
A4: $\frac{\partial ARE_{GCLM}}{\partial t}$	$k_{b_{46}}$ = 14.6 $zmol^{-3}.s^{-1}$	$k_{u_{46}}$ = 0.02 s^{-1}	n_{46} = 3
		$ARE_{GCLM_{(initial)}}$ = 6.35.E^{-4} zmol	
A5: $\frac{\partial ARE_{GS}}{\partial t}$	$k_{b_{32}}$ = 0.29 $zmol^{-2}.s^{-1}$	$k_{u_{32}}$ = 0.02 s^{-1}	n_{32} = 2
		$ARE_{GS_{(initial)}}$ = 6.35E^{-4} zmol	
A6: $\frac{\partial ARE_{GST}}{\partial t}$	$k_{b_{54}}$ = 0.29 $zmol^{-2}.s^{-1}$	$k_{u_{54}}$ = 0.02 s^{-1}	n_{54} = 2
		$ARE_{GST_{(initial)}}$ = 6.35E^{-4} zmol	
A7: $\frac{\partial ARE_{GPx}}{\partial t}$	$k_{b_{54b}}$ = 0.29 $zmol^{-2}.s^{-1}$	$k_{u_{54b}}$ = 0.02 s^{-1}	n_{54b} = 2
		$ARE_{GPx_{(initial)}}$ = 6.35E^{-4} zmol	
A8: $\frac{\partial ARE_{MRP}}{\partial t}$	$k_{b_{63}}$ = 0.29 $zmol^{-2}.s^{-1}$	$k_{u_{63}}$ = 0.02 s^{-1}	n_{63} = 2
		$ARE_{MRP_{(initial)}}$ = 6.35E^{-4} zmol	
A9: $\frac{\partial ARE_{Nrf2}}{\partial t}$	$k_{b_{25}}$ = 5.26E^{-3} $zmol^{-1}.s^{-1}$	$k_{u_{25}}$ = 0.02 s^{-1}	n_{25} = 1
		$ARE_{Nrf2_{(initial)}}$ = 6.35E^{-3} zmol	
A10: $\frac{\partial ARNT_{Nucleus}}{\partial t}$	k_{b_6} = 0.014 $zmol^{-1}.s^{-1}$	k_{u_6} = 0.002 s^{-1}	$ARNT_{(initial)}$ = 6.35E^{-3} zmol

Table S1(followed)

Equation	Parameters and initial state variables
A11: $\frac{\partial CsA_{cytosol}}{\partial t}$ A12: $\frac{\partial CsA_{extracellar}}{\partial t}$ A13: $\frac{\partial CsA_{nuleus}}{\partial t}$ A14: $\frac{\partial CsA_{wall}}{\partial t}$	$V_{cytosol}$ = 1702 µm^3 ; CL_{in_1} = 99.6 zmol.sec^{-1} ; CL_{out_1} = 1483 zmol.sec^{-1} ; $V_{nucleus}$ = 380 µm^3 ; Km_{out_1} = 2965 µmol.L^{-1} ; k_3 = 0.921 ; $CsA_{cytosol(initial)}$ = 0 zmol ; V_{max_2} = 0.2 sec^{-1} ; Km_2 = 2.18E^6 zmol ; $CsA_{wall(initial)}$ = 0 zmol ; $CL_{in_4} = CL_{in_1}$; $CL_{out_4} = CL_{out_1}$; $CsA_{nucleus(initial)}$ = 0 zmol ; k_1 = 3.55E^{-5} sec^{-1} ; k_2 = 6.01E^{-4} zmol$^{(1-k3)}$.sec^{-1} ; Refer to A1 and A2 for the other parameters.
A15: $\frac{\partial CsA\_AhR_{cytosol}}{\partial t}$	$CsA\_AhR_{cytosol(initial)}$ = 0 zmol ; CL_{in_3} = 10 µm^3.s^{-1} ; CL_{out_3} = 1 µm^3.sec^{-1} ; Refer to A1 for the other parameters.
A16: $\frac{\partial CsA\_AhR_{nucleus}}{\partial t}$	$CsA\_AhR_{nucleus(initial)}$ = 0 zmol ; Refer to A2, A11 and A15 for the parameters.
A17: $\frac{\partial CYP_{cytosol}}{\partial t}$	$CYP_{cytosol(initial)}$ = 34 zmol ; $k_{deg_{24}}$ = 1E-4 s^{-1} ; $k_{TSL_{23}}$ = 0041 s^{-1}
A18: $\frac{\partial DRE_{CYP}}{\partial t}$	$k_{b_{19}}$ = 1.39 zmol^{-2}.s^{-1} ; $k_{u_{19}}$ = 0.018 s^{-1} ; n_{19} = 2 ; $DRE_{CYP(initial)}$ = 6.35E^{-4} zmol
A19: $\frac{\partial DRE_{GST}}{\partial t}$	$k_{b_{55}}$ = 13.9 zmol^{-2}.s^{-1} ; $k_{u_{55}}$ = 0.02 s^{-1} ; n_{55} = 2 ; $DRE_{GST(initial)}$ = 6.35E^{-4} zmol
A20: $\frac{\partial DRE_{MRP}}{\partial t}$	$k_{b_{64}}$ = 0.26 zmol^{-2}.s^{-1} ; $k_{u_{64}}$ = 0.02 s^{-1} ; n_{64} = 1 ; $DRE_{MRP(initial)}$ = 6.35E^{-4} zmol
A21: $\frac{\partial DRE_{Nrf2}}{\partial t}$	$k_{b_{26}}$ = 13.9 zmol^{-2}.s^{-1} ; $k_{u_{26}}$ = 0.02 s^{-1} ; n_{26} = 2 ; $DRE_{Nrf2(initial)}$ = 6.35E^{-4} zmol

Table S1(followed)

Equation	Parameters and initial state variables
A22: $\frac{\partial GCL_{cytosol}}{\partial t}$	$k_{b_{52}}$ = -[a] $k_{u_{52}}$ = 0.02 s^{-1} $k_{deg_{53}}$ = 3.86E^{-5} s^{-1} $GCL_{cytosol(initial)}$ = 1020 zmol
A23: $\frac{\partial GCLC_{cytosol}}{\partial t}$	$GCLC_{cytosol(initial)}$ = 2890 zmol $k_{deg_{45}}$ = 3.86E^{-5} s^{-1} $k_{TSL_{44}}$ = 0.0417 s^{-1} Refer to A22 for the other parameters.
A24: $\frac{\partial GCLM_{cytosol}}{\partial t}$	$GCLM_{cytosol(initial)}$ = 612 zmol $k_{deg_{51}}$ = 3.86E^{-5} s^{-1} $k_{TSL_{50}}$ = 0.0417 s^{-1} Refer to A22 for the other parameters.
A25: $\frac{\partial Gene_{CYP_{OFF}}}{\partial t}$ A26: $\frac{\partial Gene_{CYP_{ON}}}{\partial t}$	$Gene_{CYP_{OFF}(initial)}$ = 6.35E^{-4} zmol $k_{act_{20}}$ = 2.5E^{-5} s^{-1} $k_{desact_{20}}$ = 0.01 s^{-1} $Gene_{CYP_{ON}(initial)}$ = 0 zmol $k_{ind(NMA)_{20}}$ = 0.079 zmol^{-1}.s^{-1}
A27: $\frac{\partial Gene_{GCLC_{OFF}}}{\partial t}$ 28: $\frac{\partial Gene_{GCLC_{ON}}}{\partial t}$	$Gene_{GCLC_{OFF}(initial)}$ = 6.35E^{-4} zmol $k_{act_{41}}$ = 4E^{-4} s^{-1} $k_{desact_{41}}$ = 0.01 s^{-1} $Gene_{GCLC_{ON}(initial)}$ = 0 zmol $k_{ind_{41}}$ = -[a]
A29: $\frac{\partial Gene_{GCLM_{OFF}}}{\partial t}$ A30: $\frac{\partial Gene_{GCLM_{ON}}}{\partial t}$	$Gene_{GCLM_{OFF}(initial)}$ = 6.35E^{-4} zmol $k_{act_{47}}$ = 4E^{-4} s^{-1} $k_{desact_{47}}$ = 0.01 s^{-1} $Gene_{GCLM_{ON}(initial)}$ = 0 zmol $k_{ind(NMA)_{47}}$ = -[a]
A31: $\frac{\partial Gene_{GS_{OFF}}}{\partial t}$ A32: $\frac{\partial Gene_{GS_{ON}}}{\partial t}$	$Gene_{GS_{OFF}(initial)}$ = 6.35E^{-4} zmol $k_{act_{33}}$ = 5E^{-4} s^{-1} $k_{desact_{33}}$ = 0.01 s^{-1} $Gene_{GS_{ON}(initial)}$ = 0 zmol $k_{ind(NMA)_{33}}$ = -[a]
A33: $\frac{\partial Gene_{GST_{OFF}}}{\partial t}$ A34: $\frac{\partial Gene_{GST_{ON}}}{\partial t}$	$Gene_{GST_{OFF}(initial)}$ = 6.35E^{-4} zmol $k_{act_{56}}$ = 1E^{-3} s^{-1} $k_{desact_{56}}$ = 0.01 s^{-1} $Gene_{GST_{ON}(initial)}$ = 0 zmol $k_{ind(NMA)_{56}}$ = -[a] $k_{ind(XAAD)_{56}}$ = 0.26 zmol^{-1}.s^{-1}

[a] Estimated parameter.

Table S1(followed)

Equation	Parameters and initial state variables		
A35: $\frac{\partial Gene_{GPx_{OFF}}}{\partial t}$ A36: $\frac{\partial Gene_{GPx_{ON}}}{\partial t}$	$Gene_{GPx_{OFF}}{}_{(initial)}$ = 6.35E^{-4} zmol	$k_{act_{56b}}$ = 1E^{-3} s^{-1}	$k_{desact_{56b}}$ = 0.01 s^{-1}
	$Gene_{GPx_{ON}}{}_{(initial)}$ = 0 zmol	$k_{ind(NMA)_{56b}}$ = -[a]	
A37: $\frac{\partial Gene_{MRP_{OFF}}}{\partial t}$ A38: $\frac{\partial Gene_{MRP_{ON}}}{\partial t}$	$Gene_{MRP_{OFF}}{}_{(initial)}$ = 6.35E^{-4} zmol	$k_{act_{65}}$ = 6.8E^{-4} s^{-1}	$k_{desact_{65}}$ = 0.01 s^{-1}
	$Gene_{MRP_{ON}}{}_{(initial)}$ = 0 zmol	$k_{ind(NMA)_{65}}$ = -[a]	$k_{ind(XAAD)_{65}}$ = 1.32 zmol^{-1}.s^{-1}
A39: $\frac{\partial Gene_{Nrf2_{OFF}}}{\partial t}$ A40: $\frac{\partial Gene_{Nrf2_{ON}}}{\partial t}$	$Gene_{Nrf2_{OFF}}{}_{(initial)}$ = 6.35E^{-4} zmol	$k_{act_{27}}$ = 2.5E^{-3} s^{-1}	$k_{desact_{27}}$ = 0.01 s^{-1}
	$Gene_{Nrf2_{ON}}{}_{(initial)}$ = 0 zmol	$k_{ind(NMA)_{27}}$ = -[a]	$k_{ind(XAAD)_{27}}$ = 13 zmol^{-1}.s^{-1}
A41: $\frac{\partial GS_{cytosol}}{\partial t}$ A42: $\frac{\partial GSmono_{cytosol}}{\partial t}$	$k_{b_{38}}$ = 1.2E^{-4} zmol^{-1}.s^{-1}	$k_{u_{38}}$ = 0.02 s^{-1}	$k_{deg_{39}}$ = 1.93E^{-5} s^{-1}
	$GS_{cytosol}{}_{(initial)}$ = 1632 zmol	$k_{TSL_{36}}$ = 0.0417 s^{-1}	$k_{deg_{37}}$ = 3.86E^{-5} s^{-1}
	$GSmono_{cytosol}{}_{(initial)}$ = 0 zmol		

[a] Estimated parameter.

Table S1(followed)

Equation	Parameters and initial state variables		
A43-A51: $\frac{\partial \gamma GC_{cytosol}}{\partial t}$	$Gly_{cytosol}$ = 1.7E6 zmol	$K_{m1_{73}}$ = 11.2E^{5} zmol	$K_{m2_{73}}$ = 2.6E^{6} zmol
	$ATP_{cytosol}$ = 8.5E6 zmol	$Glu_{cytosol}$ = 1.7E7 zmol	$Cys_{cytosol}$ = 5.1E5 zmol
	$v_{max_{73}}$ = -[a]	$K_{m(Gly)_{73}}$ = 3E^{6} zmol	$K_{m(ATP)_{73}}$ = 12E^{4} zmol
	$K_{m(Glu)(GCL)_{72}}$ = 8.2E^{5} zmol	$v_{max(GCL)_{72}}$ = -[a]	$v_{max(GCLC)_{72}}$ = -[a]
	$\gamma GC_{cytosol_{(initial)}}$ = 2.6E^{5} zmol	$K_{m(ATP)(GCLC)_{72}}$ = 8.5E^{6} zmol	$K_{is(ATP)(GCLC)_{72}}$ = 2.2E^{6} zmol
	$K_{m(ATP)(GCL)_{72}}$ = 14.8E^{5} zmol	$K_{ii(ATP)(GCLC)_{72}}$ = 6.8E^{5} zmol	$K_{m(Glu)(GCLC)_{72}}$ = 2.7E^{6} zmol
	$K_{is(ATP)(GCL)_{72}}$ = 11E^{6} zmol	$K_{is(Glu)(GCLC)_{72}}$ = 5.1E^{5} zmol	$K_{ii(Glu)(GCLC)_{72}}$ = 14E^{5} zmol
	$K_{ii(ATP)(GCL)_{72}}$ = 6.6E^{6} zmol	$K_{m(Cys)(GCL)_{72}}$ = 3.7E^{5} zmol	$K_{m(Cys)(GCLC)_{72}}$ = 4.6E^{5} zmol
	$K_{is(Glu)(GCL)_{72}}$ = 14E^{5} zmol	$K_{ii(Glu)(GCL)_{72}}$ = 5.3E^{6} zmol	
A52: $\frac{\partial GSH_{cytosol}}{\partial t}$	$GSH_{cytosol_{(initial)}}$ = 8.5E^{6} zmol	$v_{max_{74}}$ = 3137 zmol.s^{-1}	$K_{m_{74}}$ = 3.4E^{7} zmol
	$K_{i(GSH)_{8b}}$ = 14.5E^{4} zmol	$K_{m(GSH)_{8b}}$ = 8.5E^{5} zmol	$v_{max_{8b}}$ = -[a]
	$K_{m(ROS)_{8b}}$ = 8.5E^{4} zmol	$K_{i(ROS)_{8b}}$ = 14.5E^{4} zmol	Refer to A51 for the other parameters.
A53: $\frac{\partial GST_{cytosol}}{\partial t}$ A54: $\frac{\partial GSTmono_{cytosol}}{\partial t}$	$k_{b_{61}}$ = 3.4E^{-4} zmol.s^{-1}	$k_{u_{61}}$ = 0.02 s^{-1}	$k_{deg_{62}}$ = 1.29E^{-5} s^{-1}
	$GST_{cytosol_{(initial)}}$ = 250 zmol	$k_{TSL_{59}}$ = 0.0417 s^{-1}	$k_{deg_{60}}$ = 1.29E^{-4} s^{-1}
	$GSTmono_{cytosol_{(initial)}}$ = 206 zmol		
A55: $\frac{\partial Keap1_{cytosol}}{\partial t}$ A56: $\frac{\partial Keap1o_{cytosol}}{\partial t}$	$Keap1_{cytosol_{(initial)}}$ = 34 zmol	$k_{red_{10}}$ = 0.1 s^{-1}	$k_{ox_{10}}$ = -[a]
	$Keap1o_{cytosol_{(initial)}}$ = 0 zmol	$k_{b_{14}}$ = 3.4E^{-3} zmol.s^{-1}	$k_{u_{14}}$ = 0.02 s^{-1}
	$k_{u_{12}}$ = 0.014 s^{-1}	$k_{b_{15}}$ = 3.4E^{-3} zmol.s^{-1}	$k_{u_{15}}$ = 0.02 s^{-1}
	$k_{u_{13}}$ = 1E^{-4} s^{-1}		

[a] Estimated parameter.

Table S1(followed)

Equation	Parameters and initial state variables		
A57: $\frac{\partial mRNA_{CYP}}{\partial t}$	$mRNA_{CYP\,(initial)}$ = 0.787 zmol	$k_{TSP_{21}}$ = -[a]	$k_{deg_{22}}$ = 6E^{-5} s^{-1}
A58: $\frac{\partial mRNA_{GCLC}}{\partial t}$	$mRNA_{GCLC\,(initial)}$ = 3.08 zmol	$k_{TSP_{42}}$ = -[a]	$k_{deg_{43}}$ = 4.83E^{-5} s^{-1}
A59: $\frac{\partial mRNA_{GCLM}}{\partial t}$	$mRNA_{GCLM\,(initial)}$ = 1.33 zmol	$k_{TSP_{48}}$ = -[a]	$k_{deg_{49}}$ = 4.83E^{-5} s^{-1}
A60: $\frac{\partial mRNA_{GS}}{\partial t}$	$mRNA_{GS\,(initial)}$ = 0.731 zmol	$k_{TSP_{34}}$ = -[a]	$k_{deg_{35}}$ = 4.83E^{-5} s^{-1}
A61: $\frac{\partial mRNA_{GST}}{\partial t}$	$mRNA_{GST\,(initial)}$ = 0.731 zmol	$k_{TSP_{57}}$ = -[a]	$k_{deg_{58}}$ = 4.71E^{-5} s^{-1}
A62: $\frac{\partial mRNA_{GPx}}{\partial t}$	$mRNA_{GPx\,(initial)}$ = 0.731 zmol	$k_{TSP_{57b}}$ = -[a]	$k_{deg_{58b}}$ = 4.71E^{-5} s^{-1}
A63: $\frac{\partial mRNA_{MRP}}{\partial t}$	$mRNA_{MRP\,(initial)}$ = 6.46 zmol	$k_{TSP_{66}}$ = -[a]	$k_{deg_{67}}$ = 1.93E^{-5} s^{-1}
A64: $\frac{\partial mRNA_{Nrf2}}{\partial t}$	$mRNA_{Nrf2\,(initial)}$ = 0.046 zmol	$k_{TSP_{28}}$ = -[a]	$k_{deg_{29}}$ = 6.43E^{-5} s^{-1}
A65: $\frac{\partial MRP_{cytosol}}{\partial t}$ A66: $\frac{\partial MRPmono_{cytosol}}{\partial t}$	$k_{b_{70}}$ = 0.59E^{-5} zmol^{-1}.s^{-1}	$k_{u_{70}}$ = 0.02 s^{-1}	$k_{deg_{71}}$ = 7.15E^{-6} s^{-1}
	$MRP_{cytosol\,(initial)}$ = 3.4E^{3} zmol	$k_{TSL_{68}}$ = 0.0417 s^{-1}	$k_{deg_{69}}$ = 1.93E^{-5} s^{-1}
	$MRPmono_{cytosol\,(initial)}$ = 3.4E^{3} zmol		
A67: $\frac{\partial MAF_{nucleus}}{\partial t}$	$MAF_{nucleus\,(initial)}$ = 0.038 zmol	$k_{b_{18}}$ = -[a]	$k_{u_{18}}$ = 0.02 s^{-1}
A68: $\frac{\partial NMA_{GCLC}}{\partial t}$	$NMA_{GCLC\,(initial)}$ = 0 zmol	Refer to A3 for the parameters.	
A69: $\frac{\partial NMA_{GCLM}}{\partial t}$	$NMA_{GCLM\,(initial)}$ = 0 zmol	Refer to A4 for the parameters.	
A70: $\frac{\partial NMA_{GS}}{\partial t}$	$NMA_{GS\,(initial)}$ = 0 zmol	Refer to A5 for the parameters.	
A71: $\frac{\partial NMA_{GST}}{\partial t}$	$NMA_{GST\,(initial)}$ = 0 zmol	Refer to A6 for the parameters.	
A72: $\frac{\partial NMA_{GPx}}{\partial t}$	$NMA_{GPx\,(initial)}$ = 0 zmol	Refer to A7 for the parameters.	
A73: $\frac{\partial NMA_{MRP}}{\partial t}$	$NMA_{MRP\,(initial)}$ = 0 zmol	Refer to A8 for the parameters.	
A74: $\frac{\partial NMA_{Nrf2}}{\partial t}$	$NMA_{Nrf2\,(initial)}$ = 0 zmol	Refer to A9 for the parameters.	

[a] Estimated parameter.

Table S1(followed)

Equation	Parameters and initial state variables
A75: $\frac{\partial Nrf2_{cytosol}}{\partial t}$ A76: $\frac{\partial Nrf2_{nucleus}}{\partial t}$	$Nrf2_{cytosol_{(initial)}}$ = 0.85 zmol; $k_{deg_{31}}$ = 1E^{-4} s^{-1}; $k_{TSL_{30}}$ = 0.0417 s^{-1}; $Nrf2_{nucleus_{(initial)}}$ = 0.038 zmol; $CL_{in_{16}}$ = 2 µm^3.s^{-1}; $CL_{out_{16}}$ = 1 µm^3.s^{-1}; $k_{deg_{17}}$ = 1E^{-4} s^{-1}. Refer to A55, A56, A64 and A67 for the other parameters.
A77: $\frac{\partial Nrf2\_Keap1_{cytosol}}{\partial t}$ A78: $\frac{\partial Nrf2\_Keap1o_{cytosol}}{\partial t}$	$Nrf2\_Keap1_{cytosol_{(initial)}}$ = 0 zmol; $k_{red_{11}}$ = 0.1 s^{-1}; $Nrf2\_Keap1o_{cytosol_{(initial)}}$ = 0 zmol. Refer to A55, A56, A62 and A63 for the other parameters.
A79 : $\frac{\partial Nrf2\_MAF_{nucleus}}{\partial t}$	$Nrf2\_MAF_{nucleus_{(initial)}}$ = 0 zmol. Refer to A3, A4, A5, A6, A7, A8 and A67 for the parameters.
A80: $\frac{\partial ROS_{cytosol}}{\partial t}$	$ROS_{cytosol_{(initial)}}$ = 0 zmol; $k_{f_{75}}$ = -[a]; k_{ROS} = -[a]. Refer to A52 for the other parameters.
A81: $\frac{\partial XAA_{nucleus}}{\partial t}$	$XAA_{nucleus_{(initial)}}$ = 0 zmol. Refer to A9, A17, A18, A19 and A20 for the parameters.
A82: $\frac{\partial XAAD_{CYP}}{\partial t}$	$XAAD_{CYP_{(initial)}}$ = 0 zmol. Refer to A18 for the parameters.
A83: $\frac{\partial XAAD_{GST}}{\partial t}$	$XAAD_{GST_{(initial)}}$ = 0 zmol. Refer to A19 for the parameters.
A84: $\frac{\partial XAAD_{MRP}}{\partial t}$	$XAAD_{MRP_{(initial)}}$ = 0 zmol. Refer to A20 for the parameters.
A85: $\frac{\partial XAAD_{Nrf2}}{\partial t}$	$XAAD_{Nrf2_{(initial)}}$ = 0 zmol. Refer to A21 for the parameters.

[a] Estimated parameter.

Table S2: Cyclosporine A quantities measured in the extracellular medium (3mL) at low CsA concentration exposure (5 µM).

Day	Time (in hr)	CsA quantity (zeptomol)		
		Replicate 1	Replicate 2	Replicate 3
1	0.5	5.229E-06	5.105E-06	4.667E-06
	1	4.667E-06	5.000E-06	3.924E-06
	3	5.076E-06	4.695E-06	4.657E-06
	6	4.610E-06	4.743E-06	4.343E-06
	24	4.686E-06	4.352E-06	3.810E-06
3	24	5.781E-06	5.619E-06	5.210E-06
5	24	6.010E-06	5.771E-06	6.010E-06
7	24	5.076E-06	6.048E-06	5.771E-06
10	24	5.562E-06	5.410E-06	5.181E-06
14	0.5	4.905E-06	5.962E-06	5.219E-06
	1	5.333E-06	5.610E-06	5.076E-06
	3	5.124E-06	5.314E-06	4.419E-06
	6	5.943E-06	5.562E-06	5.162E-06
	24	5.238E-06	5.295E-06	4.971E-06

Table S3: Intracellular Cyclosporine A quantities measured at low CsA concentration exposure (5 µM).

Day	Time (in hr)	CsA quantity (zeptomol)		
		Replicate 1	Replicate 2	Replicate 3
1	0.5	1.248E-06	1.181E-06	9.048E-07
	1	1.286E-06	1.152E-06	1.114E-06
	3	1.390E-06	1.362E-06	1.381E-06
	6	1.152E-06	1.248E-06	1.124E-06
	24	1.657E-06	1.305E-06	1.171E-06
14	0.5	1.771E-06	2.414E-06	2.086E-06
	1	3.271E-06	3.771E-06	3.886E-06
	3	2.600E-06	1.886E-06	2.100E-06
	6	1.914E-06	2.000E-06	2.543E-06
	24	2.043E-06	2.229E-06	2.514E-06

Table S4: Cyclosporine A quantities measured on plastic at low CsA concentration exposure (5 µM).

Day	Time (in hr)	CsA quantity (zeptomol)		
		Replicate 1	Replicate 2	Replicate 3
1	0.5	6.143E-07	7.095E-07	7.286E-07
	1	5.762E-07	6.429E-07	6.619E-07
	3	5.810E-07	7.667E-07	5.905E-07
	6	7.905E-07	8.333E-07	8.095E-07
	24	7.714E-07	1.024E-06	1.019E-06
14	0.5	1.010E-06	9.810E-07	8.952E-07
	1	9.048E-07	9.905E-07	1.062E-06
	3	1.414E-06	1.229E-06	1.462E-06
	6	9.238E-07	9.429E-07	8.762E-07
	24	7.524E-07	6.333E-07	8.000E-07

Table S5: Cyclosporine A quantities measured in the extracellular medium (3 mL) at high CsA concentration exposure (15 µM).

Day	Time (in hr)	CsA quantity (zeptomol)		
		Replicate 1	Replicate 2	Replicate 3
1	0.5	1.888E-05	2.057E-05	1.693E-05
	1	1.686E-05	1.733E-05	1.636E-05
	3	1.557E-05	1.788E-05	1.674E-05
	6	1.555E-05	1.583E-05	1.745E-05
	24	1.581E-05	1.660E-05	1.490E-05
3	24	1.664E-05	1.355E-05	1.538E-05
5	24	1.674E-05	1.729E-05	1.605E-05
7	24	1.519E-05	1.612E-05	1.419E-05
10	24	1.652E-05	1.683E-05	1.757E-05
14	0.5	1.917E-05	2.076E-05	1.962E-05
	1	2.033E-05	2.007E-05	1.893E-05
	3	1.855E-05	1.886E-05	1.833E-05
	6	1.836E-05	1.850E-05	1.843E-05
	24	1.643E-05	1.612E-05	1.717E-05

Table S6: Intracellular Cyclosporine A quantities measured at high CsA concentration exposure (15 µM).

Day	Time (in hr)	CsA quantity (zeptomol)		
		Replicate 1	Replicate 2	Replicate 3
1	0.5	2.010E-06	1.671E-06	1.619E-07
	1	1.852E-06	2.086E-06	1.762E-06
	3	2.643E-06	2.500E-06	2.029E-06
	6	2.643E-06	2.310E-06	2.548E-06
	24	3.714E-05	2.548E-06	3.429E-06
14	0.5	2.222E-05	2.191E-05	1.696E-05
	1	2.160E-05	2.303E-05	2.371E-05
	3	1.350E-05	1.647E-05	1.548E-05
	6	1.603E-05	1.727E-05	2.315E-05
	24	1.467E-05	1.356E-05	1.418E-05

Table S7: Cyclosporine A quantities measured on plastic at high CsA concentration exposure (15 µM).

Day	Time (in hr)	CsA quantity (zeptomol)		
		Replicate 1	Replicate 2	Replicate 3
1	0.5	4.190E-07	5.238E-07	6.857E-07
	1	6.429E-07	8.782E-07	8.857E-07
	3	1.533E-07	9.000E-07	9.810E-07
	6	7.810E-07	1.143E-06	1.105E-06
	24	7.143E-07	1.090E-06	6.381E-07
14	0.5	5.905E-06	4.552E-06	5.000E-06
	1	6.390E-06	6.181E-06	7.067E-06
	3	8.752E-06	7.905E-06	4.848E-06
	6	6.248E-06	4.829E-06	4.448E-06
	24	5.086E-06	6.305E-06	5.762E-06

Table S8: Fold changes measured at low CsA concentration (5 µM).

Species	Day 1			Day 3			Day 14		
	R1	R2	R3	R1	R2	R3	R1	R2	R3
CYP mRNA	1.115	/	/	1.050	/	/	0.945	/	/
GCLC mRNA	1.043	1.046	/	1.093	1.038	/	1.022	0.995	/
GCLM mRNA	0.999	0.897	/	1.198	1.169	/	1.168	1.316	/
GPx mRNA	0.909	/	/	0.930	/	/	0.685	/	/
GS mRNA	0.951	/	/	0.886	/	/	0.971	/	/
GST mRNA	1.069	1.054	/	0.948	0.981	/	1.081	1.033	/
MRP mRNA	1.086	/	/	1.093	/	/	1.133	/	/
Nrf2 mRNA	0.935	/	/	1.329	/	/	1.156	/	/
GCLM	/	/	/	1.069	/	/	/	/	/
GS	0.949	1.010	1.020	0.952	0.964	1.017	0.971	1.003	1.040
GSH	1.092	1.702	0.701	1.416	0.799	1.428	1.945	0.932	1.468
γ-GC	4.602	39.391	2.869	5.192	1.538	2.135	0.542	2.749	0.232
MRP	/	/	/	/	/	/	0.782	/	/

Table S9: Fold changes measured at high CsA concentration (15 μM).

Species	Day 1			Day 3			Day 14		
	**R1**	**R2**	**R3**	**R1**	**R2**	**R3**	**R1**	**R2**	**R3**
CYP mRNA	1.099	/	/	1.004	/	/	1.211	/	/
GCLC mRNA	3.425	1.299	/	1.945	1.211	/	2.478	1.206	/
GCLM mRNA	2.755	4.709	/	2.848	5.154	/	1.769	2.785	/
GS mRNA	0.764	/	/	0.728	/	/	0.816	/	/
GPx mRNA	4.622	/	/	2.478	/	/	1.476	/	/
GST mRNA	1.056	1.077	/	1.010	0.980	/	0.986	0.974	/
MRP mRNA	1.317	/	/	1.194	/	/	1.081	/	/
Nrf2 mRNA	1.133	/	/	1.408	/	/	1.261	/	/
GCLM	/	/	/	1.431	/	/	/	/	/
GS	0.971	1.035	0.973	0.955	0.988	1.040	0.943	0.980	1.007
GSH	2.093	1.423	4.528	7.063	8.376	4.216	5.017	2.899	6.975
γ-GC	30.13	181.4	11.38	92.085	525.6	32.53	14.237	39.93	6.953
MRP	/	/	/	/	/	/	0.775	/	/

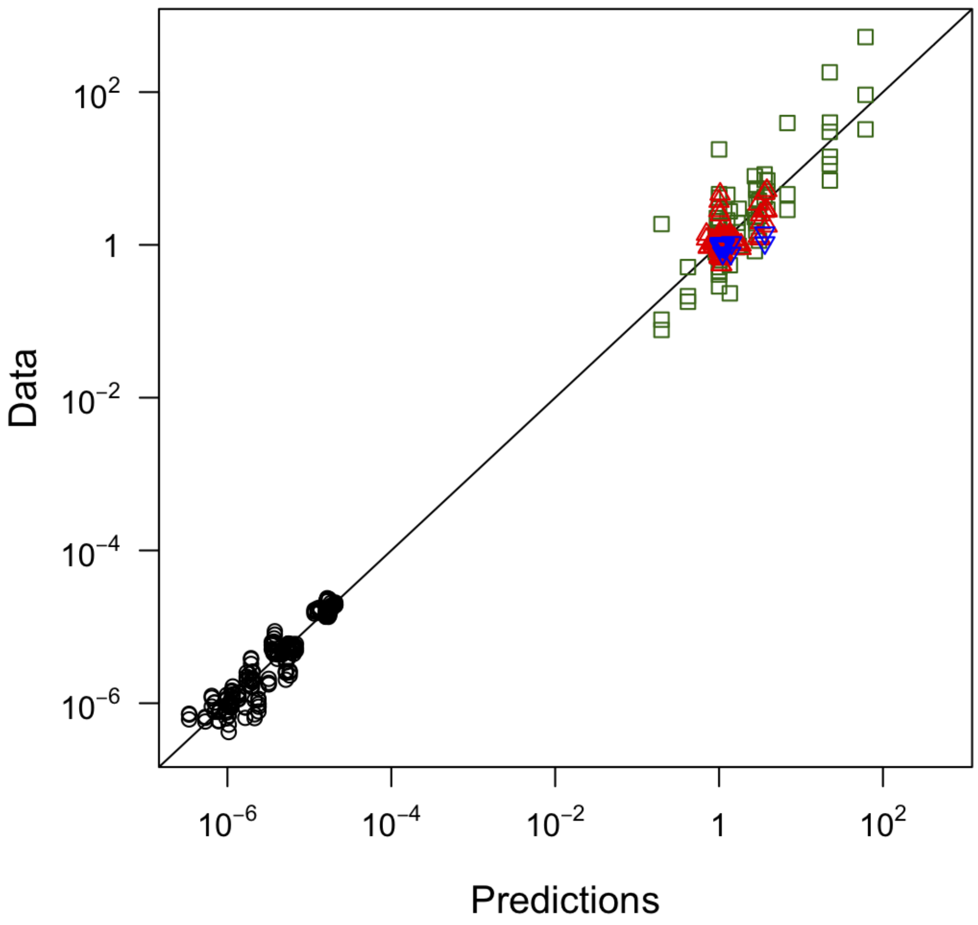

Figure S3: Model fit to the data. The data values are plotted against the model predictions, after model calibration. The PK data are represented by black circles, the metabolomic data by green square, transcriptomic by red triangles and proteomics by blue inverted triangles. For a "perfect" fit (unexpected given the measurement errors in the data and the model approximations) all points would fall on the diagonal. The average relative error is about 38%.

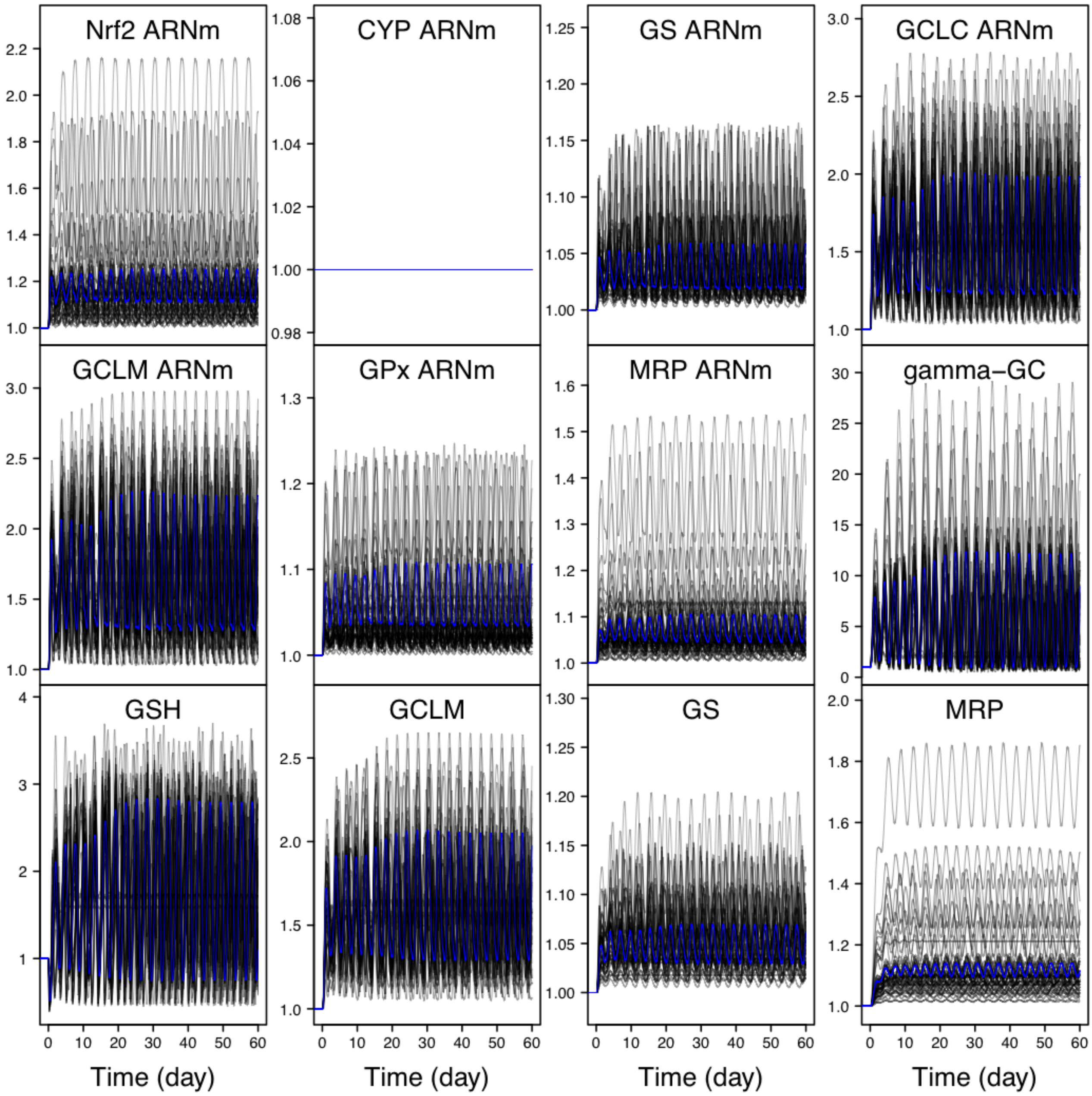

Figure S4: Transcriptomics (Nrf2 mRNA, CYP mRNA, GS mRNA, GCLC mRNA, GCLM mRNA, GPx mRNA, and MRP mRNA) proteomics (GCLM, GS, and MRP), and metabolomics (γ-GC, and GSH) fold-changes time-course in RPTEC cells during 60 days with repeated low dose (5 μM) CsA dosing. The blue line indicates the best fitting (maximum posterior probability) model prediction. The black lines are predictions made with 49 random parameter sets.

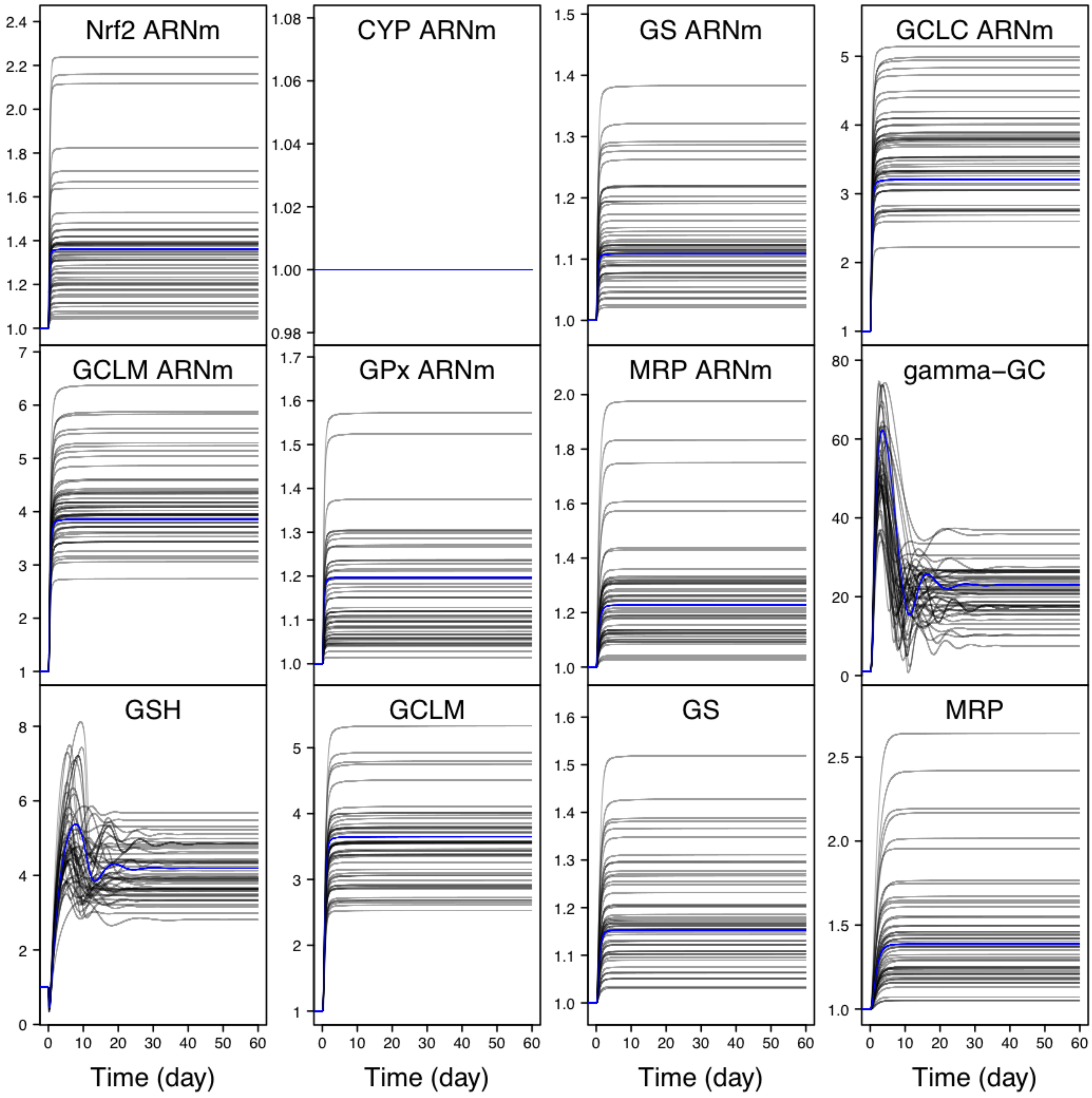

Figure S5: Transcriptomics (Nrf2 mRNA, CYP mRNA, GS mRNA, GCLC mRNA, GCLM mRNA, GPx mRNA, and MRP mRNA) proteomics (GCLM, GS, and MRP), and metabolomics (γ-GC, and GSH) fold-changes time-course in RPTEC cells during 60 days with repeated high dose (15 µM) CsA dosing. The blue line indicates the best fitting (maximum posterior probability) model prediction. The black lines are predictions made with 49 random parameter sets.
